# First investigation of pathogenic bacteria, protozoa and viruses in rodents and shrews in context of forest-savannah-urban areas interface in the city of Franceville (Gabon)

**DOI:** 10.1371/journal.pone.0248244

**Published:** 2021-03-08

**Authors:** Joa Braïthe Mangombi, Nadine N’dilimabaka, Jean-Bernard Lekana-Douki, Octavie Banga, Sydney Maghendji-Nzondo, Mathieu Bourgarel, Eric Leroy, Florence Fenollar, Oleg Mediannikov

**Affiliations:** 1 Centre Interdisciplinaire de Recherches Médicales de Franceville (CIRMF), Franceville, Gabon; 2 Aix Marseille Univ, IRD, AP-HM, Microbes, VITROME, Marseille, France; 3 IHU Méditerranée Infection, Marseille, France; 4 Département de Biologie, Faculté des sciences, Université des Sciences et Techniques de Masuku (USTM), Franceville, Gabon; 5 Département de Parasitologie, Université des Sciences de la Santé (USS), Owendo, Libreville; 6 Département Epidémiologie-Biostatistique et Informatique Médicale (DEBIM), Université des Sciences de la Santé (USS), Owendo, Libreville; 7 CIRAD, UMR ASTRE, Harare, Zimbabwe; 8 ASTRE, Univ Montpellier, CIRAD, INRA, Montpellier, France; 9 UMR MIVEGEC IRD-CNRS-UM, IRD, Montpellier, France; 10 Aix Marseille Univ, IRD, AP-HM, Microbes, MEPHI, Marseille, France; University of Pretoria, SOUTH AFRICA

## Abstract

Rodents are reservoirs of numerous zoonotic diseases caused by bacteria, protozoans, or viruses. In Gabon, the circulation and maintenance of rodent-borne zoonotic infectious agents are poorly studied and are often limited to one type of pathogen. Among the three existing studies on this topic, two are focused on a zoonotic virus, and the third is focused on rodent *Plasmodium*. In this study, we searched for a wide range of bacteria, protozoa and viruses in different organs of rodents from the town of Franceville in Gabon. Samples from one hundred and ninety-eight (198) small mammals captured, including two invasive rodent species, five native rodent species and 19 shrews belonging to the *Soricidae* family, were screened. The investigated pathogens were bacteria from the *Rickettsiaceae* and *Anaplasmataceae* families, *Mycoplasma* spp., *Bartonella* spp., *Borrelia* spp., *Orientia* spp., *Occidentia* spp., *Leptospira* spp., *Streptobacillus moniliformis*, *Coxiella burnetii*, and *Yersinia pestis;* parasites from class *Kinetoplastida* spp. (*Leishmania* spp., *Trypanosoma* spp.), *Piroplasmidae* spp., and *Toxoplasma gondii*; and viruses from *Paramyxoviridae*, *Hantaviridae*, *Flaviviridae* and *Mammarenavirus* spp. We identified the following pathogenic bacteria: *Anaplasma* spp. (8.1%; 16/198), *Bartonella* spp. (6.6%; 13/198), *Coxiella* spp. (5.1%; 10/198) and *Leptospira* spp. (3.5%; 7/198); and protozoans: *Piroplasma* sp. (1%; 2/198), *Toxoplasma gondii* (0.5%; 1/198), and *Trypanosoma* sp. (7%; 14/198). None of the targeted viral genes were detected. These pathogens were found in Gabonese rodents, mainly *Lophuromys* sp., *Lemniscomys striatus* and *Praomys* sp. We also identified new genotypes: *Candidatus* Bartonella gabonensis and Uncultured *Anaplasma* spp. This study shows that rodents in Gabon harbor some human pathogenic bacteria and protozoans. It is necessary to determine whether the identified microorganisms are capable of undergoing zoonotic transmission from rodents to humans and if they may be responsible for human cases of febrile disease of unknown etiology in Gabon.

## Introduction

For several decades, rodents have been recognized as reservoirs or hosts carrying zoonotic pathogens [1–4] that can have very dramatic impacts on the economy and public health [2]. These zoonoses include the plague [5, 6], Lassa hemorrhagic fever (LHF) [7], and hemorrhagic fever with renal syndrome [8]. Even today, zoonotic diseases involving rodents may cause hundreds or even thousands of deaths worldwide [9–11]. Many of these rodent-borne diseases are often misdiagnosed. For example, leptospirosis cases can easily be misdiagnosed as dengue or malaria infection because of the similarity of the initial symptoms [12]. Such misdiagnosis is especially frequent in countries of sub-Saharan Africa, where access to the necessary diagnostic tools is limited [13, 14].

Countries of sub-Saharan Africa are experiencing a remarkable expansion of their urban agglomerations [15–18]. This growth of cities is so dramatic that it may exceed the absorption and management capacity of municipal environmental services, leading to the development of large informal areas characterized by particularly degraded socioenvironmental conditions (high human density, waste accumulation, precarious dwellings, etc.) [18–20]. Such living conditions are reported risk factors favorable to rodent infestations [21], leading to the assertion that the level of infestation of cities by rodents is correlated with the rapid growth of these cities [22]. Finally, when the density of a rodent population increases, contacts (direct or indirect) between rodents and humans will become more common, and the likelihood of disease transmission will increase [20, 23, 24].

In developing countries in sub-Saharan Africa, the contribution of rodents to human disease is very poorly understood [13, 14]. The relevant studies that have been reported to date have often focused on specific major pathogen agents such as Lassa virus, which causes thousands of cases and deaths each year in West Africa [25], or plague, which is widely studied in Madagascar, where new cases are still recorded every year [26]. However, West Africa is an exception to this situation. Indeed, numerous studies have been conducted in Senegalese rodents addressing topics ranging from rodent ecology to invasive rodents as well as the bacterial, parasitic and virus communities carried by these rodents [13, 27–35]. Similarly, studies on the ecology of rodents and rodent-borne diseases in urban areas are emerging in Niger [14, 36–38], Mali [39] and Benin [40–44].

In Gabon, located at the equator in the western portion of the Central Africa, there are many circulating pathogens. These pathogens include the etiologic agents responsible for viral hemorrhagic fever, examples Chikungunya fever, Dengue fever and Ebola hemorrhagic fever, reviewed by Bourgarel et *al*, [45]. Several diseases of parasitic origin are also reported in the region, such as toxoplasmosis [46] and malaria, which is the most common parasitosis in tropical Africa, [47–50] and shows no improvement compared to other countries in the region according to the most recent data [51]. Many such pathogens may be carried by rodents, but in Gabon, very few studies have focused on inventories or the identification of potentially zoonotic infectious agents in these animals. The existing studies have focused on one virus at a time [52, 53] and one plasmodium parasite [54] in rodents. They do not provide sufficient data to reveal the diversity and abundance of infectious agents carried by rodents in Gabon.

Franceville is the third largest city in Gabon. It is located in the southeast of the country and is characterized by a spatial structure in which constructed, forest and savannah areas come into contact, referred to as a forest mosaic and savannah [55]. This heterogeneity of habitats makes Franceville city an excellent model for the study of zoonoses since the human population is in close contact with both domestic and wild animals in this area.

In this study, we sought to identify a wide range of potential zoonotic bacteria, protozoans and viruses hosted by rodents in the city of Franceville. The aims were to (i) identify and characterize these pathogens and (ii) compare their distribution according to the different types of habitats encountered within the city. This is the first time such a study has been conducted on rodents in Gabon.

## Materials and methods

### Franceville, study area

Franceville is a Gabonese city located 500 km southeast of the capital, Libreville. Its population increased dramatically between 1993 and 2013, from 31,193 to 110,568 inhabitants [56, 57]. It continues to grow at a moderate rate; the current population is approximately 129,000 [58]. Franceville is an atypical city characterized by the presence of buildings and vegetation referred to as mosaic forest and savannah.

**Fig 1:**
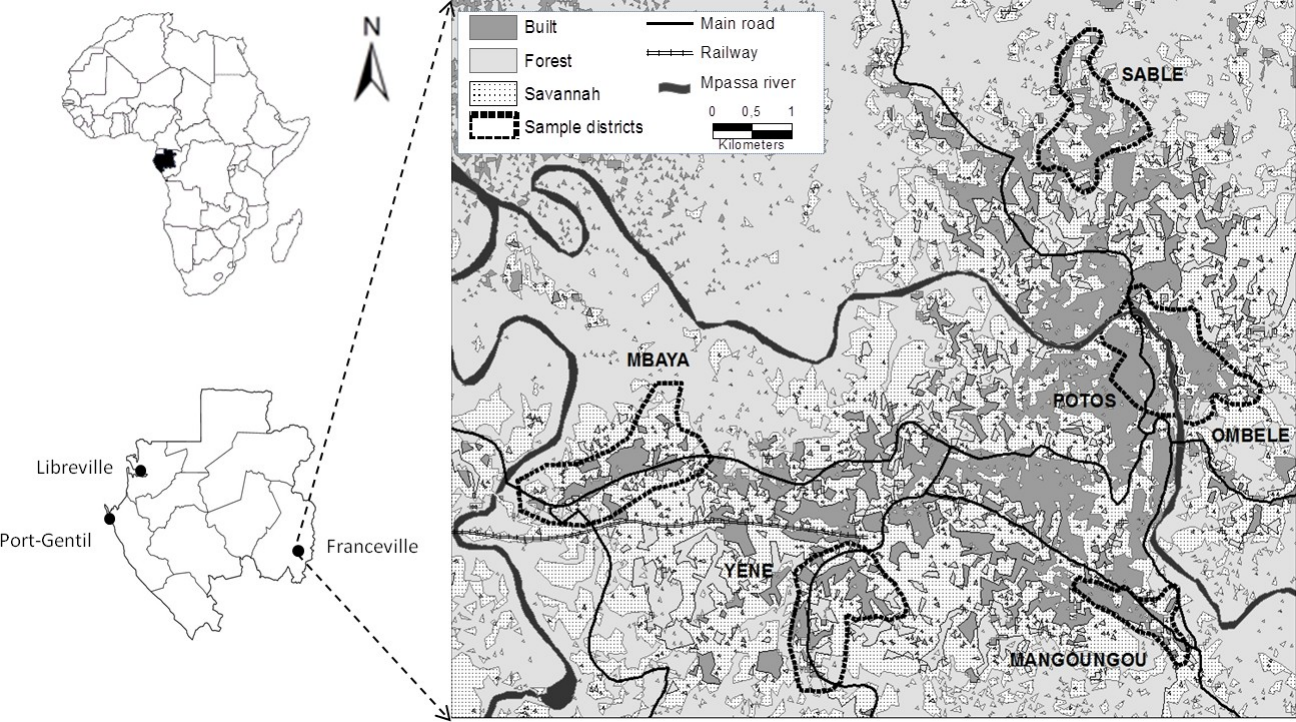
Map of Franceville. Study area and location of the six districts sampled for rodents.

Rodents were captured during four trapping sessions in houses and small savannah and forest islands in 2014. Trapping took place in houses in six (6) districts of the city of Franceville (Fig 1), including four peripheral districts (Mangoungou, Mbaya Sable and Yéné) and two central districts (Ombélé and Potos). It should also be noted that these districts are located along the main access routes to the city (roads and railways). These districts display many of the dominant characteristics of the city as a whole in terms of the level of connectivity and the aggregation of buildings as well as the presence or absence of vegetation. Mbaya and Yéné are the two main entry points of the city, by road and railway, respectively. Sable and Mangoungou are more isolated districts. Mbaya is mainly industrial. Potos is the central trade district, including large storehouses and the main open market [59].

### Rodent and organ sampling

Rodents were sampled according to a standardized live-trapping protocol as previously described [59]. Live-trapped rodents were brought back to our laboratory, euthanized, weighed, sexed, measured and autopsied. During autopsy, various organs and tissues, including the kidney, liver, brain, lungs, and spleen, were collected. All of these samples were stored at -80°C. In this study, all bacteria and protozoa were screened on the liver, except *Leptospira* on the kidney and *Toxoplasma* on the brain. While, all viruses were screened in the spleen.

### Ethics statements

Trapping campaigns were performed with prior (oral) agreement from local authorities (city Mayor, district chief and family heads). All sampling procedures were approved by the Ethics Committee named “Comité Nationale d’Ethique pour la Recherche” under the number: Prot n° 0020/2013/SG/CNE. Live-trapped rodents were brought back to our laboratory, euthanized with a halothane solution and autopsied in accordance with guidelines of the American Society of Mammalogists [60]. None of the rodent species captured in the present study had a protected status (CITES lists and IUCN).

### Specific identification of rodent species

Specific species identification was carried out according to the identification keys provided by Jean-Marc DUPLANTIER and Violaine NICOLAS following their studies on rodents in Gabon [61–69].

*Rattus rattus* was the only rat species identified in our study. Morphologically, it is easily distinguishable from *Rattus norvegicus*. In addition, the *Rattus* sample collected in this study was the subject of a population genetic study validating the identification of *R*. *rattus* [59]. Similarly, the *Mus musculus domesticus* specimens included in this study were genetically identified previously [53]. *Lemniscomys striatus* and *Lophuromys sikapusi* were identified to the species level by using morphological identification keys. *Praomys* and *Mus (Nannomys)* were identified to the subgenus level. Nevertheless, the determination of host species was performed only in pathogen-positive samples. A fragment of the 16S ribosomal RNA gene (16S rRNA) was amplified as previously described [61] and sequenced under BigDyeTM terminator cycling conditions. All sequences from this study have been deposited in GenBank under accession numbers MT256376 to MT256385 for rodent host species and MT677677 to MT677695 for shrews host species.

### DNA and RNA extraction from organs and tissues

Small pieces of the liver, kidney, spleen and brain of rodents were collected and placed individually in Eppendorf tubes. Total DNA or RNA was extracted with a BioRobot EZ1 system (Qiagen, Courtaboeuf, France) using a commercial EZ1 DNA/RNA Tissue Kit (Qiagen) (Qiagen, Courtaboeuf, France) following the manufacturer’s instructions. DNA and RNA were eluted in 100 μl of TE buffer. DNA was stored at 4°C until being used for PCR amplification, while RNA was stored at -20°C.

### Molecular detection of virus, bacterial and protozoan DNA in rodents and shrews

#### Virus molecular detection

The following virus families or species were screened by one-step RT-PCR in RNA extracts from rodent spleens: *Hantavirus* spp., *Mammarenavirus* spp., *Flavivirus*, *Paramyxovirus*, *Lymphocytic choriomeningitis mammarenavirus* (LCMV) and *Zika virus* (ZIKV). Methodological details and primer sequences are provided in Table 1.

**Table 1:** Method of investigation of targeted viruses.

Virus familly	Target virus (group)	Technique	Target gene	Primer names	Sequences (5’-3’)	Amplification	Amplicon	References
*Arenaviridae*	*Mammarenavirus*	*Nested PCR	*S*	ARS16V	GGCATWGANCCAAACTGATT	95°C for 2min, then 40 cycles of 95°C—30 S, 55°C-30 S and 72°C -1 min. Extension 72°C - 5min. **Same for the both round**	640 bp	[70]
				ARS1	CGCACCGGGGATCCTAGGC			
				ARS3V	CATGACKMTGAATTYTGTGACA		460 bp	
				ARS7C (modified)	ATRTGYCKRTGWGTTGG			
*Arenaviridae*	*LCMV*	qRT-PCR	*GPC*	LCMVS	GGGATCCTAGGCTTTTTGGAT	95°C -20 sec, then 45cycles of 95°C-3 sec 57°C- 30 sec		[53]
				LCMVAS	GCACAATAATGACAATGTTGAT			
				LCMVP- FAM	CCTCAAACATTGTCACAATCTGACCCAT			
*Hantaviridae*	*Hantavirus*	*Conventionnal PCR	*S*	UHantaF2	GGVCARACWGCHGAYTGG	95°C 2 m, then 45 cycles of 95°C -15 S, 52°C—30 S and 72°C -1m. 72°C -1 m	236 bp	[71]
				UHantaR2	TCITCWGCYTTCATDSTDGA			
			*L*	HAN-L-F2	TGCWGATGCHACIAARTGGTC	95°C- 5 m, then 45 cycles of 96°C-30 S, 60°C- 35 S and 72°C -50 S. finish with 72°C- 5min.	388 bp	[72]
				HAN-L-R2	GCRTCRTCWGARTGRTGDGCAA			
*Paramyxoviridae*	*Paramyxovirus (Respirovirus*, *Morbillivirus*, *Henipavirus)*	One step RT-PCR / semi-nested PCR	*L*	RMH-F1	TCITTCTTTAGAACITTYGGNCAYCC	60°C- 1 min for denaturing, 44 to 50°C- 30 min (for RT), 94°C- 2 min, and then 40 cycles of 94°C- 15 s, 48 to 50°C—30 s, 72°C- 30 s, and final extension 72°C-7 min **For semi-nested:** 94°C- 2 min, and then 40 cycles of 94°C- 15 s, 48 to 50°C—30 s, 72°C- 30 s, and final extension 72°C-7 min	497 bp	[73]
				RMH-F2	GCCATATTTTGTGGAATAATHATHAAYGG			
				RMH-R	CTCATTTTGTAIGTCATYTTNGCRAA			
	*Paramyxovirus (Avulavirus*, *Rubulavirus)*	One step RT-PCR / semi-nested PCR	*L*	AR-F1	GGTTATCCTCATTTITTYGARTGGATHCA		250 bp	[73]
				AR-F2	ACACTCTATGTIGGIGAICCNTTYAAYCC			
				AR-R	GCAATTGCTTGATTITCICCYTGNAC			
*Pneumoviridae*	*Paramyxovirus (Pneumovirinae)*	One step RT-PCR / semi-nested PCR	*L*	PNE-F1	GTGTAGGTAGIATGTTYGCNATGCARCC		300 bp	[73]
				PNE-F2	ACTGATCTIAGYAARTTYAAYCARGC			
				PNE-R	GTCCCACAAITTTTGRCACCANCCYTC			
*Flaviviridae*	*Zika virus*	One step RT-qPCR	*NS5*	ZIKV 1086	CCGCTGCCCAACACAAG	95°C -20 sec, then 45cycles of 95°C-3 sec 57°C- 30 sec	160 bp	[74]
				ZIKV 1162c	CCACTAACGTTCTTTTGCAGACAT			
				ZIKV 1107-FAM	AGCCTACCTTGACAAGCAGTCAGACACTCAA			
*Flaviviridae*	*Flavivirus*	One step RT-PCR / semi-nested PCR	*NS5*	PF1S	TGYRTBTAYAACATGATGGG	45°C-20 min, 95°C- 2 min,then45 cycles of 95°C- 25 sec, 51°C-30 sec, 68°C- 30 sec. End 68°C-5 min Semi-nested: 94°C- 2 min,then45 cycles of 94°C- 25 sec, 5°C-30 sec, 72°C- 30 sec. End 72°C-5 min	280 bp 210 bp	[75]
				PF3S	ATHTGGTWYATGTGGYTDGG			
				PF2Rbis	GTGTCCCAiCCNGCNGTRTC			

Oligonucleotide sequences of the primers and probes used for virus detection in rodent spleens in this study.

* Analyses were performed with cDNA using Superscript III following the manufacturer’s instructions.

#### Bacteria and protozoan molecular detection

The real-time PCR (qPCR) was performed to screen all rodent samples using previously reported primers and probes for *Bartonella* spp., *Anaplasmataceae*, *Coxiella burnetii*, *Borrelia* spp., *Rickettsiaceae*, *Mycoplasma* spp., *Orientia* spp., *Occidentia* spp., *Yersinia pestis*, *Leptospira* spp., *Streptobacillus moniliformis*., *Piroplasmida*., *Toxoplasma gondii*., and *Kinetoplastida* (including the *Trypanosoma* and *Leishmania* genera). The sequences of the primers and probes are shown in Table 2. For all systems, any sample with a cycle threshold (Ct) value of less than 40 Ct was considered positive. Conventional PCR analysis was performed for all qPCR-positive samples using the primers and conditions described in Table 2. The amplification reaction was conducted in a final volume of 25 μl containing 12.5 μl of AmpliTaq Gold master mix, 0.75 μl of each primer [20 μM], 5 μl of DNA template, and 6 μl of water. The thermal cycling profile consisted of one incubation step at 95°C for 15 min, 45 cycles of 30 s at 95°C, 30 s to 1 min at the annealing temperature (Table 2) and 1 min at 72°C, and a final extension step of 5 min at 72°C. Successful amplification was confirmed by electrophoresis in a 1.5% agarose gel, and the amplicons were completely sequenced on both strands.

**Table 2:** Method of investigation of targeted bacteria and protozoa.

Target Organism	Target gene	Technique	Primer names	SEQUENCES (5’-3’)	Annealing Temperature	Amplicon	Reference
***Anaplasmatacae***	23S	Broad-range qPCR	TtAna_F	TGACAGCGTACCTTTTGCAT	55	190 bp	[76]
			TtAna_R	GTAACAGGTTCGGTCCTCCA			
			TtAna_P	6FAM- GGATTAGACCCGAAACCAAG			
		Broad-range conventional PCR	Ana23S-212F	ATAAGCTGCGGGGAATTGTC	58	650 bp	[76]
			Ana23S-753R	TGCAAAAGGTACGCTGTCAC(for sequencing only)			
			Ana23S-908r	GTAACAGGTTCGGTCCTCCA			
***Bartonella sp***	*ITS (Intergenic 16S-23S)*	Broad-range qPCR	Barto_ITS3_F	GATGCCGGGGAAGGTTTTC	60	104 bp	[77]
			Barto_ITS3_R	GCCTGGGAGGACTTGAACCT			
			Barto_ITS3_P	6FAM- GCGCGCGCTTGATAAGCGTG			
		Broad-range conventional PCR	Urbarto1	CTTCGTTTCTCTTTCTTCA	50	733 bp	[78]
			Urbarto2	CTTCTCTTCACAATTTCAAT			
***Coxiella burnetii***	*IS1111A*	Broad-range qPCR	CB_IS1111_0706F	CAAGAAACGTATCGCTGTGGC	60	154 bp	[79]
			CB_IS1111_0706R	CACAGAGCCACCGTATGAATC			
			CB_IS1111_0706P	6FAM- CCGAGTTCGAAACAATGAGGGCTG			
	*IS30A*	Broad-range qPCR	CB_IS30A_3F	CAAGAAACGTATCGCTGTGGC	60	154 bp	[77]
			CB_IS30A_3R	CACAGAGCCACCGTATGAATC			
			CB_IS30A_3P	6FAM- CCGAGTTCGAAACAATGAGGGCTG			
	Spacer 2	Species-specific PCR	Cox2 F	CAACCCTGAATACCCAAGGA	59	358 bp	[80]
			Cox2 R	GAAGCTTCTGATAGGCGGGA			
	Spacer 5	Species-specific PCR	Cox5 F	CAGGAGCAAGCTTGAATGCG	59	344 bp	[80]
			Cox5 R	TGGTATGACAACCCGTCATG			
	Spacer 18	Species-specific PCR	Cox18 F	CGCAGACGAATTAGCCAATC	59	556 bp	[80]
			Cox18 R	TTCGATGATCCGATGGCCTT			
	Spacer 22	Species-specific PCR	Cox22 F	GGGAATAAGAGAGTTAGCTCA	59	340 bp	[80]
			Cox22 R	CGCAAATTTCGGCACAGACC			
	Spacer 37	Species-specific PCR	Cox37 F	GGCTTGTCTGGTGTAACTGT	59	322 bp	[80]
			Cox37 R	ATTCCGGGACCTTCGTTAAC			
***Leptospira sp***	*16S*	Broad-range qPCR	Lepto_F	CCCGCGTCCGATTAG	58	88 bp	[81]
			Lepto_R	TCCATTGTGGCCGRACAC			
			Lepto_P	6FAM- CTCACCAAGGCGACGATCGGTAGC			
	*LipL32*	Broad-range conventional PCR	LipL32 F	ATCTCCGTTGCACTCTTTGC	58	474 bp	[82]
			LipL32 R	ACCATCATCATCATCGTCCA			
***Borrelia sp***	*23S*	Broad-range qPCR	TTB23s F	CGATACCAGGGAAGTGAAC	60	148 bp	[78, 83]
			TTB23sR	ACAACCCYMTAAATGCAACG			
			TTB23SP	6-FAM-TTTGATTTCTTTTCCTCAGGG-TAMRA			
***Mycoplasma sp***	*ITS*	Broad-range qPCR	Mycop_ITS_F	GGGAGCTGGTAATACCCAAAGT	60	114 bp	[84]
			Mycop_ITS_R	CCATCCCCACGTTCTCGTAG			
			Mycop_ITS_P	6FAM- GCCTAAGGTAGGACTGGTGACTGGGG			
***Rickettsia sp***	gltA (CS)	Broad-range qPCR	RKND03_F	GTGAATGAAAGATTACACTATTTAT	60	166 bp	[77, 79]
			RKND03_R	GTATCTTAGCAATCATTCTAATAGC			
			RKND03 P	6-FAM- CTATTATGCTTGCGGCTGTCGGTTC			
***Orientia_Occidentia sp***	23S	Broad-range qPCR	OcOr23S-F	TGGGTGTTGGAGATTTGAGA	55	140	This study
			OcOr23S-R	TGGACGTACCTATGGTGTACCA			
			OcOr23S-P	FAM-GCTTAGATGCATTCAGCAGTT			
***Occidentia sp***	sca	Broad-range qPCR	OMscaA-F	AGTTTAAAATTCCCTGAACCACAATT	55	240	[78]
			OMscaA-R	ACTTCCAAACACTCCTGAAACTATACTTG			
			OMscaA-P	FAM-TGAAGTTGAAGATGTCCCTAATAGT			
***Streptobacillus moniliformis***	*gyrB*	Broad-range qPCR	Smoni-gyrB-F	AGTTTAAAATTCCCTGAACCACAATT	60	96 bp	[85]
			Smoni-gyrB-R	ACTTCCAAACACTCCTGAAACTATACTTG			
			Smoni-gyrB-P	6FAM-TCACAAACTAAGGCAAAACTTGGTTCATCTGAG			
***Yersina pestis***	*caf1*	species-specific qPCR	YPcaf-S	TACGGTTACGGTTACAGCAT	45	240bp	[86]
			YPcaf-A	GGTGATCCCATGTACTTAACA			
			YPcaf-1	6-FAM-ACCTGCTGCAAGTTTACCGCCTTTGG			
***Toxoplasma gondii***	ITS1	Broad-range qPCR	Tgon_ITS1_F	GATTTGCATTCAAGAAGCGTGATAGTA	60	333 bp	[87]
			Tgon_ITS1_R	AGTTTAGGAAGCAATCTGAAAGCACATC			
			Tgon_ITS1_P	6-FAM-CTGCGCTGCTTCCAATATTGG			
***Piroplasma sp***	*5*,*8S*	Broad-range qPCR	5,8s-F5	AYYKTYAGCGRTGGATGTC	60	40 bp	[78]
			5,8s-R	TCGCAGRAGTCTKCAAGTC			
			5,8s-S	6-FAM-TTYGCTGCGTCCTTCATCGTTGT			
	*18S*	Broad-range conventional PCR	piro18S-F3	GTAGGGTATTGGCCTACCG	58	969 bp	
			piro18S-R3	AGGACTACGACGGTATCTGA			
***Leishmania sp***	*18S SSU*	Broad-range qPCR	F	GGTTTAGTGCGTCCGGTG	60	75 bp	[88]
			R	ACGCCCCAGTACGTTCTCC			
			Probe leish S	FAM- CGGCCGTAACGCCTTTTCAACTCA			
***Kinetoplastidea***	*28S LSU (24 alpha)*	Broad-range qPCR	F LSU 24a	AGTATTGAGCCAAAGAAGG	60	200 bp	[88]
			R LSU 24a	TTGTCACGACTTCAGGTTCTAT			
			P LSU 24a	6FAM- TAGGAAGACCGATAGCGAACAAGTAG			
		Broad-range conventional PCR	F2 28S	ACCAAGGAGTCAAACAGACG	58	~ 550 bp	[88]
			R1 28S	ACGCCACATATCCCTAAG			
***Trypanosoma sp***	*5*. *8 S rRNA*	Broad-range qPCR	F 5,8S	CAACGTGTCGCGATGGATGA	60	83 bp	[88]
			R 5,8S	ATTCTGCAATTGATACCACTTATC			
			P 5,8S	6-FAM-GTTGAAGAACGCAGCAAAGGCGAT			
	28S	Broad-range conventional PCR	F2 28S	ACCAAGGAGTCAAACAGACG	58	~ 550 bp	[88]
			R1 28S	ACGCCACATATCCCTAAG			

Oligonucleotide sequences of primers and probes used for real‑time PCR and conventional PCR to screen bacteria and protozoans in this study.

Quantitative real-time PCR was performed on the CFX96 Real-Time system (Bio-Rad) with the Roche LightCycler 480 Probes Master Mix PCR kit (Roche Applied Science, Mannheim, Germany). For each assay, DNA extracts of the targeted bacteria or parasites were used as positive controls and distilled water as negative control (S1 Table). For the viral families *Bunyaviridae* and *Arenaviridae*, the positive controls used were plasmids, designed during the PREDICT project. For *Flaviviridae*, we used the Yellow fever virus RNA (vaccinal strain 17D) and RNA transcripts from mumps, measles, and respiratory syncytial viruses, for *Paramyxoviridae*. Conventional PCR was performed in an automated DNA thermal cycler (GeneAmp PCR Systems Applied Biosystems, Courtaboeuf, France). Sequencing analyses were performed on the ABI Prism 3130XL Genetic Analyzer (Applied Biosystems, Thermo Fisher Scientific, France) using the DNA sequencing BigDye Terminator V3.1 Cycle Sequencing Kit (Applied Biosystems, Foster City, CA, USA, Perkin-Elmer) according to the manufacturer’s instructions. The BigDye products were purified on Sefadex G-50 Superfine gel filtration resin (Cytiva, Formerly GE Healthcare Life Science, Sweden).

The sequences were compared to sequences available in the GenBank database using the BLAST algorithm (http://blast.ncbi.nlm.nih.gov/Blast.cgi).

#### Multispacer sequence typing (MST) genotyping of *Coxiella burnetii*

The multispacer sequence typing (MST) method was used for *Coxiella burnetii* genotyping. For this purpose, five (5) different spacers, which were previously described [80], were selected and amplified (Cox 2, 5, 18, 22, 37). Conventional PCR was performed as described below with a hybridization temperature of 59°C. Then, the web-based MST database (https://ifr48.timone.univ-mrs.fr/mst/coxiella_burnetii/groups.html) was used for MST identification.

### Phylogenetic and statistical analyses

#### Phylogenetic analysis

The obtained sequences were analyzed using ChromasPro version 1.3 (Technelysium Pty, Ltd., Tewantin, Queensland, Australia) for assembly and were aligned with other sequences of targeted bacteria or parasite species from GenBank using CLUSTALW, implemented in BioEdit v7.2 [89]. Phylogenetic trees were constructed with MEGA software v.7 [90]. The maximum likelihood method based on the Hasegawa-Kishino-Yano model (HKY) was used to infer the phylogenetic analysis with 500 bootstrap replicates.

#### Statistical analysis

Statistical analysis was performed with R software V3.2.5 [91] using chi-square/Fisher’s exact tests for data comparisons between the prevalence of infected rodents for all parasites according to habitat type. A *p-*value≤ 0.05 was considered to be significant.

General linear mixed models (GLMMs) run using the lme4 package [92] were also employed to examine the potential determinants of parasite richness (number of parasite species in a host), as reported in a previous similar study [78]. We assumed a Poisson distribution for the parasite richness data. The sampling site was considered a random factor, and other factors, including host factors (species, sex, weight, body mass), status (native vs. invasive), trap location (inside vs. outside the door), habitat type (central districts, peripheral districts, vegetal areas) and seasons (dry season and rainy season), were considered fixed effects (S2 and S3 Tables). The significance of the interactions of different effects was estimated by using the Akaike information criterion (AIC) for model selection. AIC changes were evaluated when model parameters were modified (added or removed). Full-model averages, available in the MuMIn package [93], were used for AIC estimation. The best model showed a null ΔAICC.

## Results

### Rodents sampled for this study

A total of 198 small mammals were captured including 49 in Mbaya, 19 in Mangoungou, 19 in Ombélé, 15 in Potos, 25 in Sable, 18 in Yéné and 53 in vegetative areas (S2 and S3 Tables). The captured animals included two (2) invasive species of rodents, *Rattus rattus* (N = 54) and *Mus musculus domesticus* (N = 29), five (5) native rodent taxa, *Lophuromys* sp. (N = 27), *Lemniscomys striatus* (N = 27), *Praomys* sp. (N = 17), *Mus* (*Nannomys*) sp. (N = 22) and *Cricetomys* sp. (N = 3), and shrews (N = 19).

According to the three types of established habitats, small mammals were distributed as follows 29 rodents (3 *Lemniscomys striatus*, 2 *M*. *m*. *domesticus*, 1 *Praomys* sp., 3 *Mus (Nannomys)* sp. and 20 *R*. *rattus*) and 5 shrews in central districts; 102 rodents (3 *Cricetomys* sp, 8 *Le*. *striatus*, 7 *Lophuromys* sp, 27 *M*. *m*. *domesticus*, 17 *Mus* (*Nannomys*) sp., 6 *Praomys* sp., 34 *R*. *rattus*) and 9 shrews in peripheral districts and 48 rodents (16 *Le*. *striatus*, 20 *Lo*. *sikapusis*, 2 *Mus* (*Nannomys*) sp., 10 *Praomys* sp.) and 5 shrews in forest-savannah areas (Table 3).

**Table 3:** Bacteria and protozoa identified in Franceville rodents.

	Pathogen screening (qPCR positive individual number)	Genotype founded	Rodent species							
			*Cricetomys* sp. (N = 3)	*Lemniscomys striatus* (N = 27)	*Lophuromys* sp. (N = 27)	*Mus m*. *domesticus*^Ŧ^ (N = 29)	*Mus Nannomys* sp. (N = 22)	*Praomys* sp. (N = 17)	*Rattus rattus*^Ŧ^ (N = 54)	*Shrews* (N = 19)
**Central districts** (Potos and Ombélé districts) **N1 = 34**	*Bartonella* spp. (1)	*Bartonella elizabethae*	0	0	0	0	0		1/54 (1.8%)	0
	*Anaplasma* spp. (1)	*Candidatus* Anaplasma gabonensis	0	0	0	0	0	0	1/54 (1.8%)	0
	*Coxiella burnetii*	-	0	0	0	0	0	0	0	0
	*Leptospira* spp.	-	0	0	0	0	0	0	0	0
	*Piroplasma*	-	0	0	0	0	0	0	0	0
	*Trypanosoma* spp. (8)	*Trypanosoma congolensis* riverine forest / *Trypanosoma brucei brucei* / *Trypanosoma otospermophili*	0	2/27 (7.4%)	0	0	1/22 (4.55%)	0	5/54 (9.3%)	0
	*Toxoplasma gondi* (1)	*Toxoplasma gondi*	0	1/27 (3.7%)	0	0	0	0	0	0
**Peripheral districts** (Mang*-Mbaya-Yéné and Sable districts) **N2 = 111**	*Bartonella* spp. (3)	*Bartonella massiliensis*	2/3 (67%)	0	1/27 (3.7%)	0	0	0	0	0
	*Anaplasma* spp.(8)	*Candidatus* Anaplasma gabonense	0	4/27 (14.8%)	0	0	0	1/17 (5.88%)	2/54 (3.7%)	1/19 (5.3%)
	*Coxiella burnetii* (3)	*Coxiella burnetii* MST group 20	0	1/27 (3.7%)	0	0	1/22 (4.55%)	0	1/54 (1.8%)	0
	*Leptospira* spp. (3)	*Lepstospira kirschneri*	0	0	0	0	0	0	1/54 (1.8%)	2/19 (10.6%)
	*Piroplasma* (1)	*Theileria* sp.	0	1/27 (3.7%)	0	0	0	0	0	0
	*Trypanosoma* spp. (6)	*Trypanosoma congolensis* riverine forest	1/3 (33%)	0	0	1/29 (3.45%)	1/22 (4.55%)	0	3/54 (5.6%)	0
	*Toxoplasma gondi*	-	0	0	0	0	0	0	0	0
**Vegetation areas** (Forest and savannah fragments) **N3 = 53**	*Bartonella* spp. (9)	*Candidatus* Bartonella gabonensis	0	0	9/27 (33.3%)	0	0	0	0	0
	*Anaplasma* spp. (7)	*Candidatus* Anaplasma gabonense	0	4/27 (14.8%)	1/27 (3.7%)	0	0	2/17 (11.8%)	0	0
	*Coxiella burnetii* (7)	*Coxiella burnetii*	0	4/27 (14.8%)	2/27 (7.4%)	0	0	1/17 (5.88%)	0	0
	*Leptospira* spp. (4)	*L*. *borgpetersenii*	0	0	4/27 (14.8%)	0	0	0	0	0
	*Piroplasma* (1)	*Theileria* sp.	0	0	0	0	0	1/17 (5.88%)	0	0
	*Trypanosoma* spp.	-	0	0	0	0	0	0	0	0
	*Toxoplasma gondi*	-	0	0	0	0	0	0	0	0

The infectious agents identified and described in this study and the rodents associated with them. One hundred and ninety-eight (198) rodents, collected in **N1**, **N2** and **N3** were analyzed by qPCR.

^Ŧ^ indicates invasive rodents.

* Mang represents the Mangoungou district.

### Bacterial, protozoan and viral nucleic acids detected in rodents and shrews

All rodents were found negative for all viral pathogens screened in the spleen by conventional PCR and qPCR, including *Hantavirus* spp., *Mammarenavirus* spp., *Flavivirus*, *Paramyxovirus*, *Lymphocytic choriomeningitis mammarenavirus* (LCMV) and *Zika virus* (ZIKV). Similarly, all rodents were found negative by qPCR on tissues for several bacteria and protozoa, specifically *Borrelia* sp, *Leishmania* sp, *Mycoplasma* sp, *Orientia* sp, *Occidentia* sp, *Streptobacillus moniliformis*, *Rickettsia* sp and *Yersinia pestis*. In contrast, 49/198 (24,7%) rodents were positive for 8 of the 16 pathogens (bacteria and protozoans) tested via qPCR. In total, 7 genera of pathogenic microorganisms were identified, including bacterial *Anaplasma* spp. (8.1%; 16/198), *Bartonella* spp. (6.6%; 13/198), *Coxiella burnetii* (5.1%; 10/198), and *Leptospira* spp. (3.5%; 7/198). The protozoans that we identified included *Trypanosoma* sp (7%; 14/198), *Piroplasma* sp (1%; 2/198) and *Toxoplasma gondii* (0.5%; 1/198) (Table 3). All microorganisms were detected in the liver samples except for *Leptospira* spp. and *Toxoplasma gondii*, which were only detected in the kidney and brain, respectively.

Multiple infections (i.e., many infectious agents in the same organ of an individual rodent) were found in 11 rodents (5.5%), including 10 double infections (5%) and one triple infection (0.5%). *Rattus rattus* and *Le*. *striatus* presented the highest carriage rate for all of the identified pathogens, including 5 out of 7 infectious agents, while *M*. *m*. *domesticus* appeared to harbor the fewest pathogens (1/7). Other species carried between 3 and 4 pathogens (Table 3).

### Phylogenetic analysis for the taxonomic description of detected pathogens

#### Bartonella

**Fig 2:**
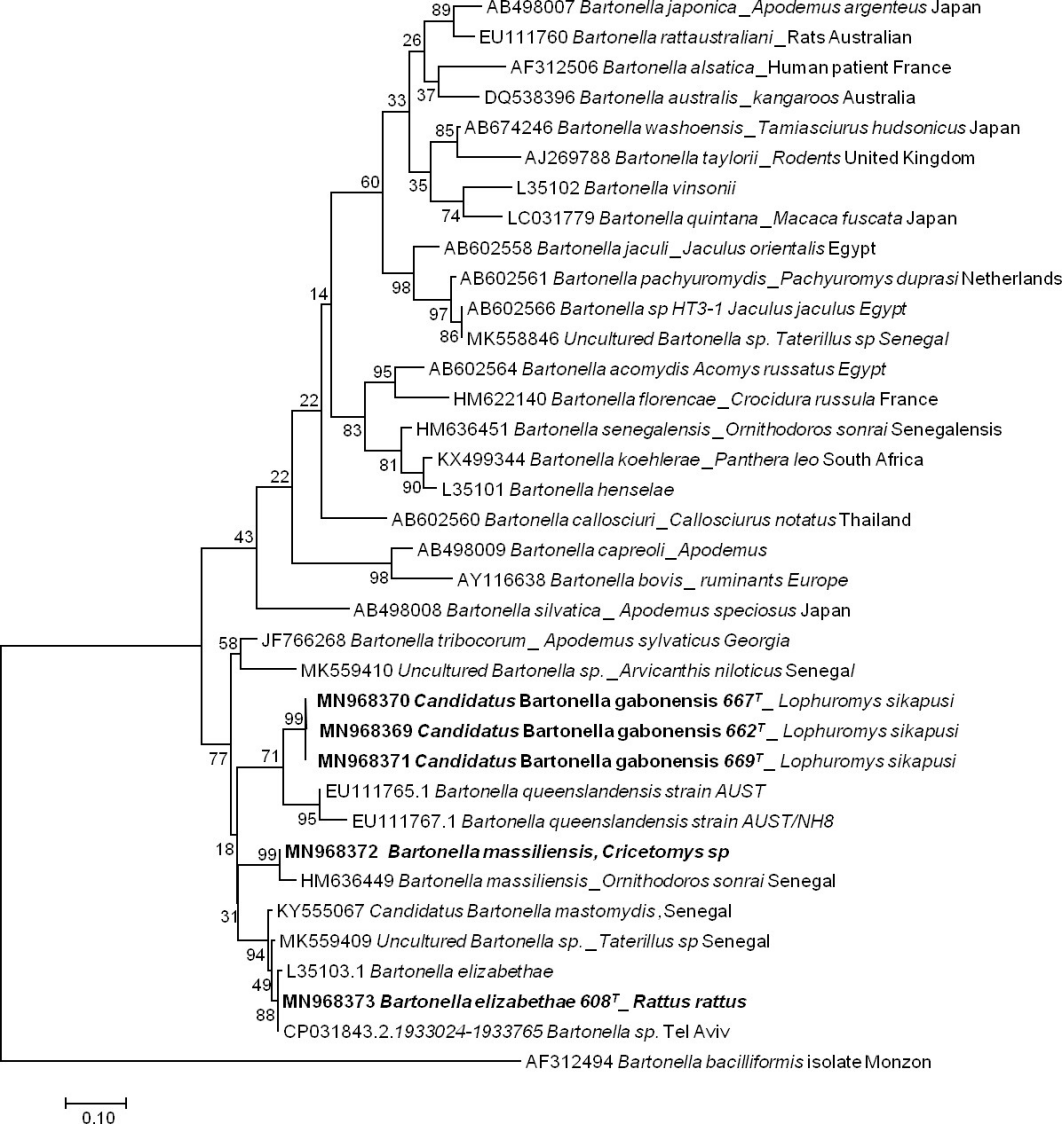
Taxonomic tree and description of the identified *Bartonella*. Phylogenetic tree of *Bartonella* spp. identified in rodents in Franceville. The evolutionary history was inferred by using the Maximum Likelihood method based on the Hasegawa-Kishino-Yano model. The analysis involved 36 nucleotide sequences. All positions containing gaps and missing data were eliminated. There were a total of 189 positions in the final dataset. Evolutionary analyses were conducted in MEGA7. Sequences obtained in this study are indicated in bold. The hosts are indicated after the underscore.

The sequencing of 733 bp of the ITS gene from the DNA extracts of 13 qPCR-positive individuals revealed five sequences of *Bartonella* ranging from 690 to 722 bp (GenBank: MN968369 to MN968373). BLAST analysis of three sequences obtained from *Lophuromys* sp. hosts showed that the most closely related species was *Bartonella queenslandensis* (GenBank: EU111769.1), which presented the highest score and a percentage of identity of 84–86% (611/721, 546/634, 550/635). This percentage of identity below 95% and the fact that all these three sequences were grouped in the same cluster (with 99% of identity between each other) in the phylogenetic tree suggested that the obtained *Bartonella* pathogen represented a new species, an undescribed species. We provisionally named this probable new genotype *Candidatus* Bartonella gabonensis. The fourth sequence obtained from a *Cricetomys* sp. rodent matched the *B*. *massiliensis* OS23 and OS09 strains (HM636450 and HM636449) with 96.7% (699/723) and 96.1% (700/728) identity, respectively. The last sequence, obtained from *R*. *rattus*, matched *Bartonella* sp. *’Tel Aviv’* of the *Bartonella elizabethae complex* (GenBank: CP031843.2) with 100% (690/690) identity. It is referred to here as *B*. *elizabethae* (Fig 2).

#### Anaplasma

**Fig 3:**
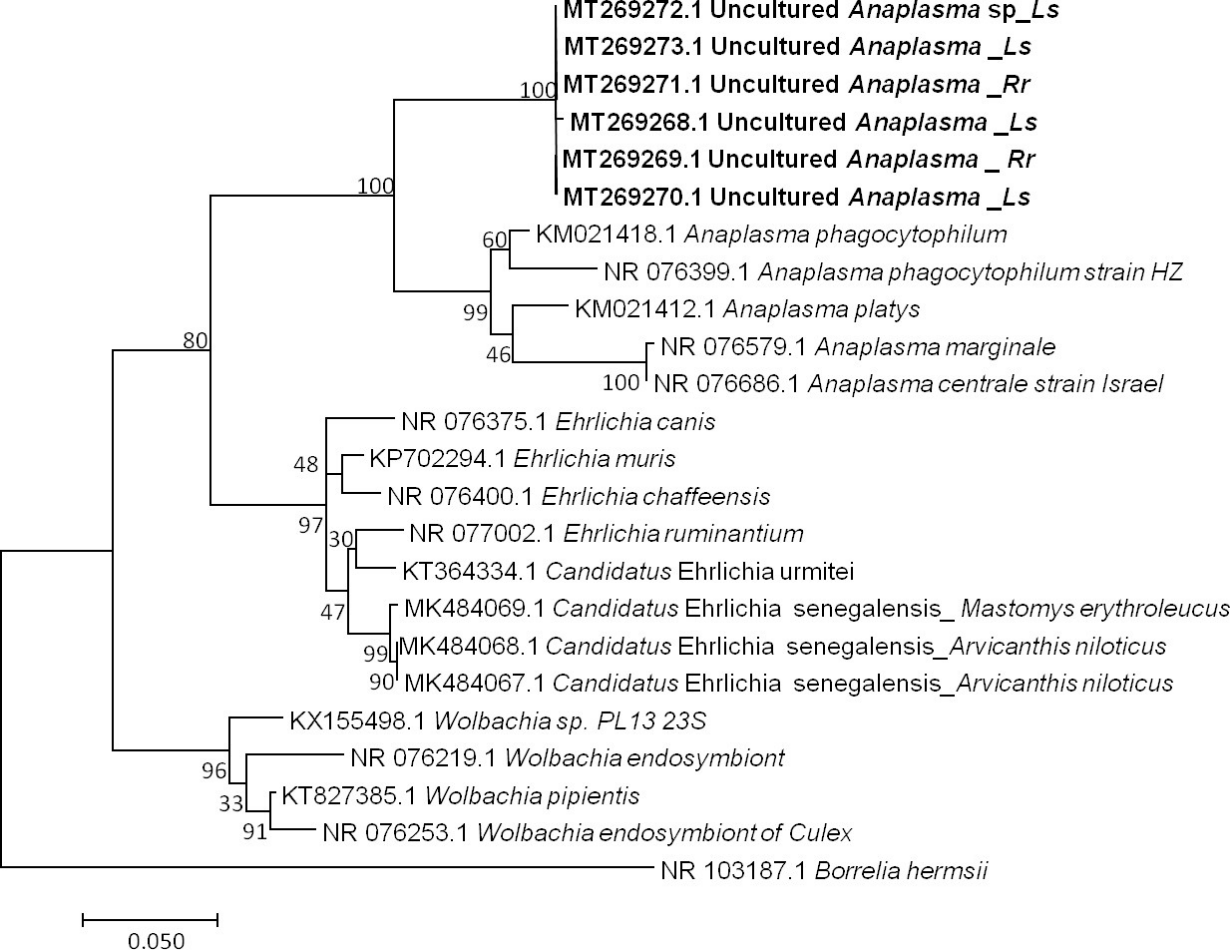
Taxonomic tree and description of the identified *Candidatus* Anaplasma gabonense. Phylogenetic tree of *Anaplasma* species identified in rodents of Franceville. The evolutionary history was inferred by using the Maximum Likelihood method based on the Hasegawa-Kishino-Yano model. The analysis involved 24 nucleotide sequences. All positions containing gaps and missing data were eliminated. There were a total of 420 positions in the final dataset. Sequences obtained in this study are indicated in bold. Evolutionary analyses were conducted in MEGA7. The hosts are indicated after the underscore. *Ls*, *Lemniscomys striatus*; *Rr*, *Rattus rattus*.

Among 16 qPCR-positive individuals, six sequences ranging from 623 to 683 bp (GenBank: MT269268 to MT269273) were obtained after the sequencing of the *Anaplasma* 23S rRNA gene [76, 94]. BLAST analysis of these sequences showed identities with *A*. *phagocytophilum* (KM021418) ranging from 91% to 92% (578/633, 606/659, 607/659, 606/660, 607/659, 607/659). The percentage of identity below 95% suggests that the obtained pathogen is a new or undescribed species, with similarity to *Anaplasma phagocytophilum*. However, the dissimilarity between the *Anaplasma* sequences, as shown in our data (Fig 3), could also suggest that the amplified genetic material would come from organisms of a different genus. Finally, not being able to conclude on the basis of our results, we refer to it here as Uncultured *Anaplasma* sp.

#### Coxiella burnetti

The genotyping of *Coxiella burnetii* from 10 qPCR-positive individuals via MST analysis showed the following profile allele codes: 3–2–6–5–4, corresponding to Cox 2—Cox5—Cox 18- Cox22—Cox37, respectively. This profile identified MST group 20. This genotype has been found in Europe and the United States [80] and is associated with human and animal disease. The same genotype, MST20 was also found on domestic animal ticks in Ethiopia in Africa [95]. In Franceville, *C*. *burnetii* MST group 20 was found in all the samples tested from the five rodent species mentioned *Praomys* sp, *R*. *rattus*, *Mus Nannomys* sp, *Le*. *striatus* and *Lo*. *sikapusi*.

#### Leptospira

Seven samples were positive in qPCR screening of the *16S* rRNA gene of *Leptospira* sp. The sequencing of a portion of the *LipL* 32 gene from the DNA extracts of 7 qPCR-positive individuals revealed two *Leptospira* sequences (MT274303 and MT274304). Sequence MT274303 was 99.1% (442/446; 444/448) similar to both *Leptospira borgpetersenii* serovar Hardjo (CP033440.1) and *Leptospira weilii* (AY461930.1), identified from *Lophuromys* sp. Sequence MT274304 was 100% (474/474) similar to both *Leptospira kirshneri* (JN683896.1) and *Leptospira interrogans* (KC800991.1), identified in the *Crocidura goliath* shrew (MT256384.1).

#### Trypanosoma

**Fig 4:**
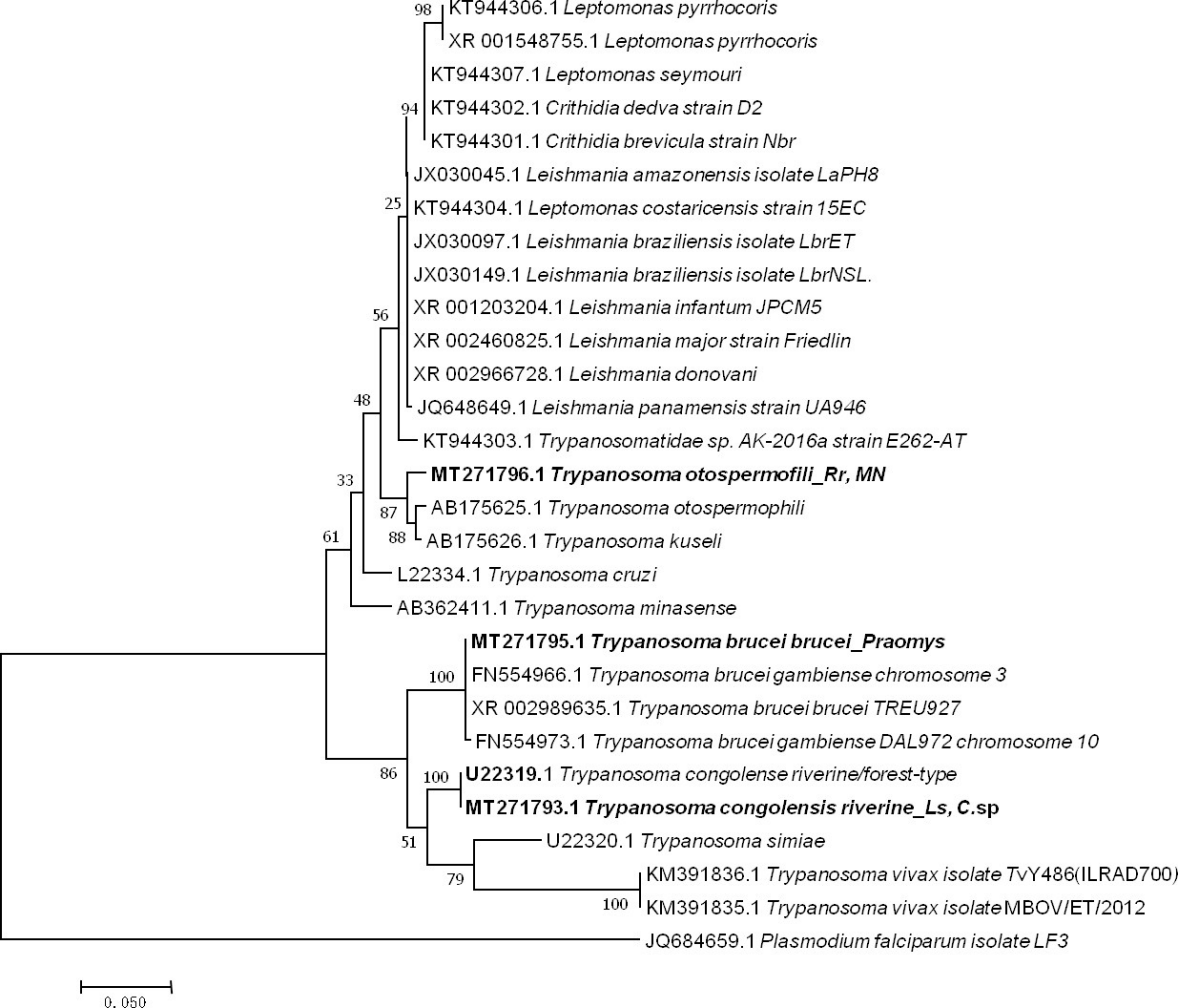
Taxonomic tree and description of the identified *Trypanosoma* sp. Maximum likelihood tree of *Trypanosoma* species identified in rodents in Franceville. Sequences obtained in this study are indicated in bold. The evolutionary history was inferred by using the Maximum Likelihood method based on the Hasegawa-Kishino-Yano model. The analysis involved 30 nucleotide sequences. All positions containing gaps and missing data were eliminated. There were a total of 415 positions in the final dataset. Evolutionary analyses were conducted in MEGA7. The hosts are indicated after the underscore. *Ls*, *Lemniscomys striatus*; *C*. sp., *Cricetomys* sp.; *MN*, *Mus Nannomys* sp.

Five sequences of *Trypanosoma* ranging from 464 to 571 bp were obtained (GenBank: MT271793 to MT271797) after the sequencing of 550bp of the *28 S* gene from 14 qPCR-positive individuals identified when screening for *Trypanosoma* spp. (10 positive individuals) and the *Kinetoplastidae* class (4 positive individuals). Two presented as *T*. *congolense riverine/forest-type* (U22319) (2/198) with 99% (570/571) of identity from *Le*. *striatus* and *Cricetomys* sp; one, from *Praomys* sp (1/198), showed 100% (522/522) identity to both *T*. *brucei brucei* (XR_002989635) and *T*. *brucei gambiense* (FN554966.1); and two others from *R*. *rattus and Mus Nannomys* (AB190228) (2/198) were identified as *T*. *otospermophili*, with 97% (453/467) identity (Fig 4).

#### Theileria

**Fig 5:**
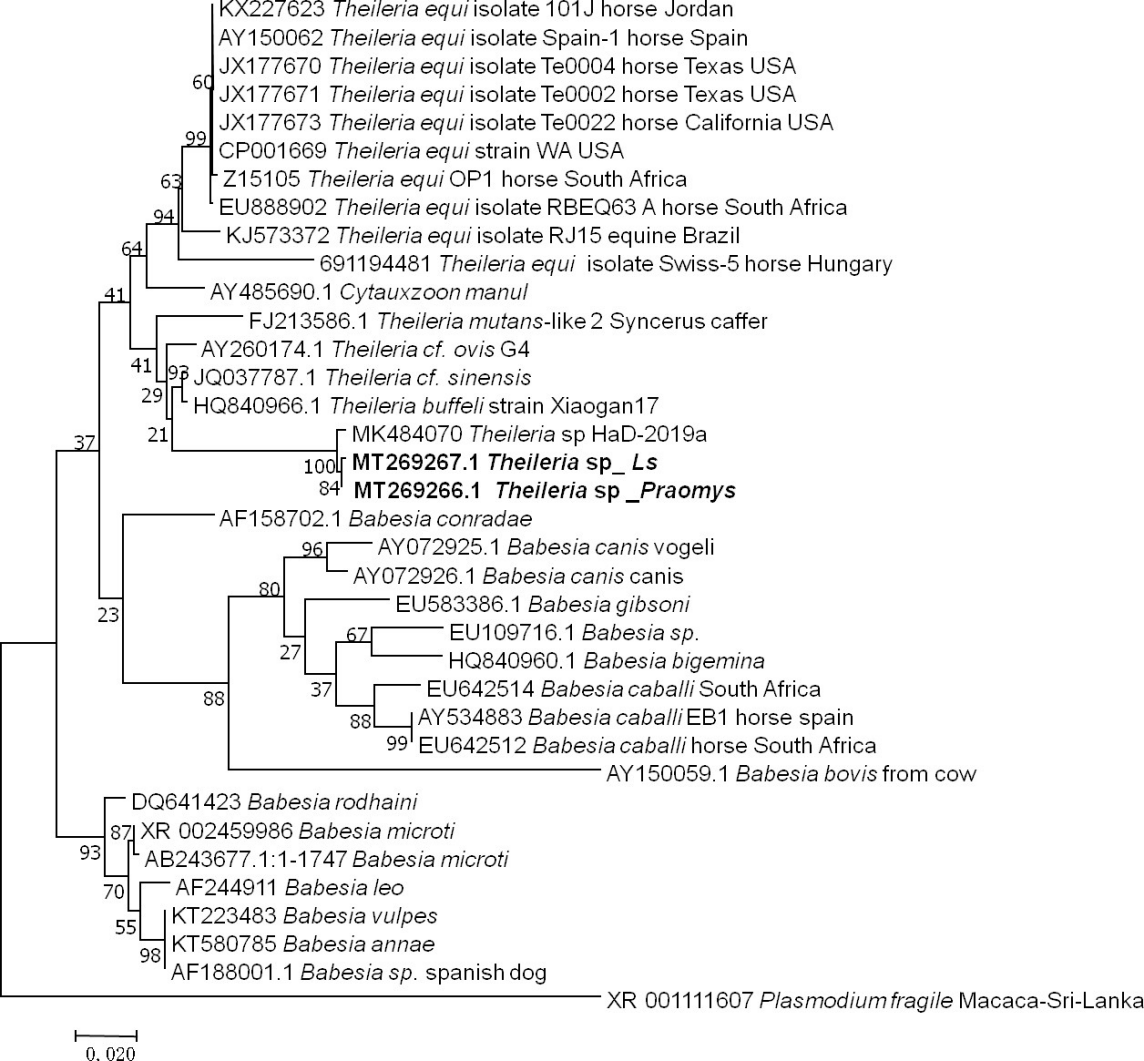
Taxonomic tree and description of the identified *Theileria* sp. Phylogenetic tree of *Theileria* species identified in rodents in Franceville. Sequences obtained in this study are indicated in bold. The evolutionary history was inferred by using the Maximum Likelihood method based on the Hasegawa-Kishino-Yano model. The analysis involved 36 nucleotide sequences. All positions containing gaps and missing data were eliminated. There were a total of 749 positions in the final dataset. Evolutionary analyses were conducted in MEGA7. *Ls*, *Lemniscomys striatus*.

The two samples that were positive according to the pan-*Piroplasma* 5.8S qPCR analysis and were sequenced (GenBank: MT269266 and MT269267) were shown 98% (869/883, 868/881) identity to *Theileria* sp strain HaD-2019a (MK484070.1) found in Senegalese rodents (Fig 5). These two infected rodents were *Le*. *striatus* and *Praomys* sp.

#### Toxoplasma gondii

Of the tested brain samples, only one (0.5%, N = 198) was positive in the qPCR screening of *T*. *gondii* according to the *ITS*1 gene, with a Ct value of 35.1. However, it could not be identified; the sample came from an *Le*. *striatus* rodent.

### Habitats and pathogens in rodents

**Fig 6:**
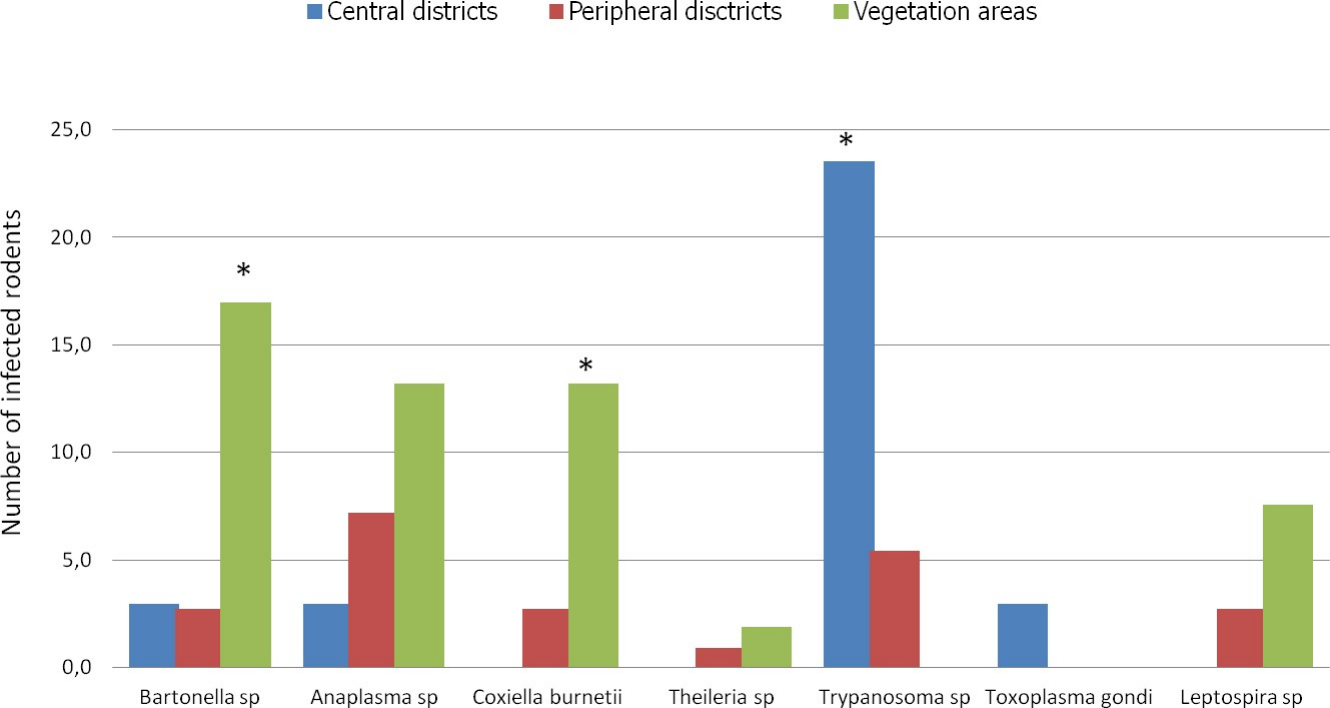
Histogram of the prevalence of infections. Richness of pathogens in Franceville rodents by habitat type. Three habitat types were identified in this study: the central district with little vegetation, peripheral districts with abundant vegetation around dwellings and vegetation areas (mixture of only savannah and forest). * indicates significantly different richness at p <0,05.

We categorized the sampling areas into three groups as follows: central districts (Ombélé, Potos), peripheral districts (Mbaya, Yéné, Sable, and Mangoungou) and non urban areas of vegetation (savannah-forest) (Fig 6). The prevalence of infection by the pathogens in each group was 32.3% (11/32), 21.6% (24/111) and 52.8% (28/53) for the central districts, peripheral districts and vegetated areas, respectively.

In terms of overall prevalence, a significant difference was found between the average prevalence in the infected rodents in the three habitat types (X-squared = 16.659, df = 2, *P* < 0.0002413). The residual value of the X-squared test showed that the difference was attributed to the vegetated areas. The rodents from vegetated areas showed the highest infection prevalence in Franceville. Similarly, at the pathogen group scale (only for pathogens found in more than 5 rodents), the rodents from vegetated areas showed the highest prevalence of infection by *Bartonella* sp *(P* <0.001) and *C*. *burneti* (*P* <0.004) pathogens (Fig 6). However, a different result was obtained in rodents from central districts, which showed a significantly higher rate of infection by *Trypanosoma* pathogens than the rodents coming from the 2 other habitats (*P*< 0.002).

### Host factors and pathogens in rodents

GLMM analysis revealed a strong association effect between the parasite richness and body mass of host rodents (*t*-value = 0.791, *P<* 0.0449) (Table 4). Parasitic richness was positively correlated with the weight of the rodents. Conversely, no association could be identified between parasitic richness and the other factors tested among the rodents in Franceville.

**Table 4:** Factors and parasitic richness association.

Fixed effects:	Estimate	SE	*t* -value	2.5%	97.5%	*p*-value
(Intercept)	0.321	0.146	2.192	0.034	0.608	0.030
Sex ^a^	0.007	0.081	0.083	-0.152	0.166	0.934
Trap location ^b^	-0.128	0.119	-1.070	-0.362	0.106	0.286
Season ^c^	-0.158	0.087	-1.821	-0.327	0.012	0.070
Weight	0.001	0.000	2.019	0.000	0.002	**0.045**
Species status ^d^	0.092	0.117	0.791	-0.136	0.321	0.430
Habitat types ^e^	0.082	0.142	0.576	-0.197	0.360	0.586

Evaluation of the Poisson generalized linear mixed models fitted to estimate the host factors and parasitic richness association.

The reference categories corresponding to:

^a^ male,

^b^ indoor,

^c^ rainy season,

^d^ native and

^e^ vegetal areas.

## Discussion

Rodents are hosts of numerous zoonotic diseases caused by bacteria, protozoans, or viruses all around the world [96]. Luis et *al*., 2013 [1] noted the importance of paying serious attention to rodents because they are the most diverse mammals and carry many pathogens responsible for emerging viral zoonoses. In Gabon, over the last five years, some studies have focused specifically on rodent viruses [52, 53] and plasmodium parasites [54]. Herein, we broaden the spectrum of these studies by investigating a wide range of bacteria, protozoa and viruses in the rodents of Franceville in Gabon.

We did not identify the presence of viruses in these rodents. Several hypotheses can be put forth to explain this result. We suggest that a low viral load could explain the failure to detect the targeted viral fragments. In such instances, the use of high-throughput sequencing, particularly next generation sequencing methods [97], could be more effective and efficient, as reported by Diagne et *al*., 2017 [27]. Another hypothesis is that the failure to detect pathogens may be due to the absence in our sample of rodent species that are reservoirs for the targeted pathogens. For example, *Mastomys natalensis* and *Mastomys erythroleucus*, which are LHF reservoirs whose distribution area includes Gabon, were missing from our sample. Nevertheless, *R*. *rattus* and *M*. *m*. *domesticus* are reservoirs of many pathogens, including *Hantavirus* and *Lymphocytic choromeningite virus* (LCMV), respectively, but we did not succeed in identifying these viruses. Nevertheless, this hypothesis is supported by the fact that these two species are invasive rodents that may lose associated viruses in new environments, referred to as the "enemy release" effect [98]. The third hypothesis that may explain the lack of virus detection is that Franceville rodents are secondary hosts for the screened viruses [99]. The studied rodents may, however, harbor many other viruses that may or may not be related to the viral species targeted in our study. Negative results in such cases can be explained by insufficiently broad primer specificity.

To obtain support for this hypothesis, serological analyses would be appropriate for determining whether the pathogen continues to circulate when nucleic acids are absent. Indeed, serological diagnoses of circulating viral infectious agents have already been carried out and shown to be effective in other studies conducted in Africa [13, 100, 101].

Our study included the first detection and description of several bacterial and protozoan species in rodent populations in Gabon. Our results revealed an overall prevalence of 25.7% (51/198) for the 7 identified microparasites. This diversity of pathogens is not surprising. Due to the location of Gabon in the Congo basin, it is expected to be a biodiversity hotspot including various pathogens [45]. In Senegal, a well-diversified bacterial community of 12 zoonotic OTUs (Operational Taxonomic Unit) was identified despite the Sahelian context and the use of different detection methods. The difference in the composition of these microparasitic communities shows the need to expand investigations to improve our knowledge of them, especially in Africa, where most studies focus on a single zoonotic agent [42, 102], except when investigating the helminth community [28, 30].

Studies on infectious agents hosted by rodents are very limited in many developing countries in sub-Saharan Africa [102], particularly in Central Africa. Existing studies have previously revealed the presence in African rodents of the bacteria and protozoans that we detected in our study. These organisms include *Bartonella* spp. [103–105], *Coxiella burnetii* [102, 106], *Anaplasma* sp. [107], *Theileria* sp. [78], *Trypanosoma* sp. [42], *Toxoplasma gondii* [108] and *Leptospira* sp. [14, 40]. The presence of these bacterial genera shows the need to explore and implement surveillance in host rodents in Gabon. These bacterial genera include species that can act as zoonotic pathogens capable of inducing severe diseases that are often misdiagnosed in Africa [79, 109, 110].

Indeed, the detected species of pathogens included *C*. *burnetii*, which constitutes a monospecific genus and is the causative agent of Q fever, which is a disease found worldwide. Zoonotic Q fever can be acute or chronic and exhibits a wide spectrum of clinical manifestations. The natural cycle of *C*. *burnetii* is not reported to include humans, who are considered incidental hosts [111]. *Coxiella burnetii* is associated with sylvatic or domestic transmission cycles, with rodents being suspected to link the two transmission cycles [102]. Consequently, human infections with *C*. *burnetii* are systematically associated with infected livestock and ticks. Nevertheless, Q fever outbreaks associated with contact with infected rodents were recently reported in Zambia [102]. We identified the *C*. *burnetii* pathogenic genotype MST 20, which was detected for the first time in central Africa but has previously been reported in an East African country, Ethiopia [95], in the United States and Europe [80]. Other known genotypes of *C*. *burnetii* in Africa are: MST 6, 19, 35 and 36 [109], discovered on a wide range of hosts, including ruminants and human febrile patients.

*Leptospira borgpetersenii*, *L*. *kirschneri*, *L*. *interrogans* and *L*. *weilii* are highly pathogenic leptospires, which are agents of leptospirosis, an emerging zoonotic disease that affects both animals and humans worldwide [112]. Based on phylogenetic analyses of DNA-DNA hybridization (DDH) and 16S rRNA data, the *Leptospira* genus has been divided into three distinct clades. The pathogenic leptospire clade includes 10 pathogens that can infect and cause disease in humans and animals [113]. In another clade, the intermediate clade, there are five leptospires that have been isolated from humans and animals that may cause various mild clinical manifestations of leptospirosis. In the third clade, the so-called saprophytes, there are seven leptospires that are unable to cause disease [112, 114]. Pathogenic *Leptospira* spp. colonize the proximal renal tubules of reservoir hosts and are excreted through urine into the external environment, where they can survive in water for several months [115].

For some time now, the incidence of tick-borne diseases in humans and animals has been increasing due to several factors that together favor the chance of contact among wild animals, their ectoparasites, domestic animals and humans [107]. The detected bacteria of the genera *Anaplasma* and *Bartonella* as well as *Theileria* sp. protozoa are responsible for tick-borne diseases. Theileriosis and anaplasmosis are mostly diseases of domestic ruminants that are responsible for economic losses to varying degrees depending on the species of the pathogen and the region of the epidemic [76, 116]. However, an increasing number of human cases of anaplasmosis (human granulocytic anaplasmosis, HGA) with *A*. *phagocytophilum* have been reported, including some fatal cases [117]. It is this species in particular that is mostly found in rodents [118–120]. Although rodents are suspected to be potential reservoirs of this bacterium, their role in the epidemiologic cycles affecting domestic and wild animals as well as humans has not been demonstrated [119]. In Africa, rodents infected with *Ehrlichia* sp. have been detected [94, 107, 121]. The most recent study revealed the probable presence of a new species of *Anaplasmatacae* infecting rodents in Senegal: *Candidatus* Ehrlichia senegalensis [78]. Among the 16 animals that were qPCR positive for *Anaplasmatacae*, six sequences were identified by 23S rRNA gene amplification, two of which came from *R*. *rattus* and four from *Le*. *striatus*. The 6 sequences were almost identical (98.72% to 99.99%). Based on the phylogenetic tree topology and the percentage of identity (91%) after BLAST analysis, our results suggest that the sequences obtained by 23S rRNA gene amplification represent an organism not yet described, close to *A*. *phagocytophylum* and could be a new species. We refer to it here as Uncultured *Anaplasma* spp. The pathogenicity of this new genotype in humans and animals is unknown.

For piroplasms, two samples were positive by qPCR, which were successfully amplified from the rodents *Le*. *striatus* and *Praomys* sp. The obtained sequences were closely related to an isolated sequence from the rodent *Arvicanthis niloticus* in Senegal designated *Candidatus* Theileria senegalensis, with 98% identity [78]. Here, we refer to it as *Theileria* sp. This is presumably the same species of bacteria isolated from different rodents, suggesting that they are not reservoirs of this bacterium; however, it could be that the source of infection is either a tick or a ruminant. To date, no human case of theileriosis has been reported; in contrast *Babesia*, particularly *Babesia microti*, has been shown to be responsible for human babesiosis, with rodents serving as hots [2, 122]. *Babesia* and *Theileria* are closely related genera within the *Piroplasmida* order.

Conversely, in the *Bartonella* genus, there are many species responsible for human diseases, such as *B*. *henselae*, causing cat scratch disease, *B*. *quintana*, causing trench fever, and *B*. *bacilliformis*, causing Carrión disease [123]. Indeed, among the 53 *Bartonella* species currently described [124], more than twenty are associated with rodents. These are the species that usually cause human infections [103]. We found three different genotypes of *Bartonella*, which were identified as *Bartonella elizabethae* from *R*. *rattus*; these genotypes were closely related to *Bartonella massiliensis* recently described [125] from *Cricetomys* sp. as well as a new genotype proposed as *Candidatus* Bartonella gabonensis from *Lo*. *sikapusi*. Thus, we identified 13 (6,6%) positive individuals with the qPCR system targeting 16S-23S rRNA, including one from *R*. *rattus*, two from *Cricetomys sp* and ten from *Lophuromys sikapusi*. Among the three genotypes that we have highlighted, the pathogenicity of two, *B*. *massiliensis* and *Candidatus* B. gabonensis, is not yet known. *B*. *elizabethae* is known to cause febrile human diseases [126], usually resulting from close contact between humans and rodents. Here, *B*. *elizabethae* was described in *R*. *rattus* captured inside a house, which implies close contact with the human inhabitant of this house and therefore an increased risk of infection with *Bartonella*, as reported [127].

*Trypanosoma* species are flagellated protozoan parasites including species that are highly pathogenic to humans, such as *T*. *cruzi*, responsible for American Chagas disease, and *T*. *brucei*, which is the causal agent of human African trypanosomiasis (HAT), also known as African sleeping sickness. Both are transmitted to humans by biting insects (*Triatominae* and tsetse flies, respectively) [128]. In rodents, many studies have revealed the presence of *T*. *lewisi*, for which rats were initially considered the only specific hosts [129]. However, *T*. *lewisi* has also been found in other rodents [38] and even in insectivores [130]. This parasite is transmissible to humans, although instances of lethal human infection have been reported in both Asia and Africa [131, 132]. In the present study, we did not find *T*. *lewisi*. However, among the 14 individuals that tested positive for *Trypanosoma* spp. by qPCR, we successful amplification was achieved from 6. Two of these sequences were identical to a *T*. *congolense* riverine/forest-type from *Le*. *striatus* and *Cricetomys* sp.; one was identical to both *T*. *brucei brucei* and *T*. *brucei gambiense* from *Praomys* sp.; and the last two were identical to *T*. *otospermophili* from *R*. *rattus* and *Mus (Nannomys)* sp. *Trypanosoma congolense* riverine/forest-type trypanosomes are the most economically important trypanosomes causing African animal trypanosomiasis (AAT) and losses in domestic animals (cattle, goats, sheep, horses, pigs and dogs) in sub-Saharan Africa [133, 134]. *Trypanosoma congolense* has been classified as savannah, riverine-forest and Kilifi types, which are morphologically identical but genetically heterogeneous types that vary in their virulence, pathogenicity, vectors and geographical distribution [135]. Nevertheless, studies have frequently identified coinfections of different *T*. *congolense* types in livestock and tsetse flies [136]. On the other hand, *Trypanosoma otospermophili* is a species hosted by rodents that is very poorly studied [137]. *Trypanosoma brucei brucei* is the only subspecies among the three that is not infectious to humans. *T*. *brucei gambiense* and *T*. *brucei rhodesiense* cause a chronic form and an acute form of sleeping sickness, respectively [128]. The identification of *T*.*brucei gambiense* reveals the risk of transmission of this pathogen from rodents to humans in Franceville. Nevertheless, a specific analysis (another gene or more variable regions) would be necessary to determine precisely which species is described here, *T*. *brucei brucei* or *T*. *brucei gambiense*.

*Toxoplasma gondii* is an intracellular protozoan responsible for toxoplasmosis, which is an anthropozoonosis that is widely distributed around the world. Domestic or wild felids are the definitive hosts of this parasite, and a wide range of terrestrial or marine mammals and birds, including rodents, are intermediate hosts [138]. We found one individual positive for *Toxoplasma* from *Le*. *striatus* by qPCR, but amplification to describe the genotype was unsuccessful. Indeed, *T*. *gondii* is a monospecific genus; however, it presents several strains whose virulence profiles are variable according to the host species [108]. This result reflects the lack of specific PCR amplification tools, which are currently being developed following the recommendations of various collaborators.

Furthermore, our results show that the prevalence of pathogens is higher in native rodents, notably species such as *Lemniscomys* sp. (55%), *Lophuromys* sp. (62%) and *Praomys* sp. that are associated with vegetation, compared to that found in the commensal invasive species *R*. *rattus* (23%) and *M*. *m*. *domesticus* (3.4%) (Table 3). This situation is similar to what was recently highlighted in Senegal [78]. It would be difficult to speculate here about the involvement of these microparasite communities in the invasion of the black rat or even the domestic mouse, as described, for example, in Senegal [27]. We do not have enough data to make such inferences.

Otherwise, the pathogen species detected and described according to phylogenetic trees (Figs 2–5) are pathogens associated with rural areas, peridomestic areas or even plants. With the exception of *Leptospira* species, *B*. *elizabaethae* and *T*. *gondii*, the other pathogens identified here, including the *Trypanosoma* species, are not infectious to humans but cause disease in domestic animals [139]. Therefore, these results highlight the relationship between vegetation and pathogens, more specifically, the implication of interactions between wildlife and domestic fauna in the circulation of infectious agents and their transmission to humans. Our results also highlight multihost pathogens, particularly *T*. *otospermophili* (Fig 4) and *Candidatus* Anaplasma gabonensis (Fig 3), which infect both *R*. *rattus* and *Mus (Nannomys)* sp. or both *R*. *rattus Praomys* sp. and *Le*. *striatus*, respectively [140].

Conversely, pathogenic agents of monospecific hosts have also been detected, as indicated by the phylogenetic tree of *Bartonella* species. The probable presence of a new species, referred to as *Candidatus* Bartonella gabonensis, was observed in the rodent species *Lo*. *sikapusi*. In addition, our results provide evidence of the circulation of new bacterial genotypes: *Candidatus* Bartonella gabonensis and Uncultured *Anaplasma* spp.

Our results concerning the pathogens *C*. *burnetii* and *Anaplasma* spp. could also correspond to the spill back hypothesis [141] because these pathogens associated with forest and savannah rodents are found in the invasive rodent *R*. *rattus*. Additional data would be required to confirm this assumption.

None of the factors analyzed here as potential determinants of parasitic richness in Franceville rodents can be questioned except for animal weight, where we found that the heaviest rodents were the most infected (Table 4), as previously reported [27, 78, 142]. Larger individuals may have a larger home range, which increases their frequency of contact with parasites [143]. In addition, since body mass can be considered an indicator of host age, the generally positive correlation between infection and body mass may reflect the longer duration of exposure in older rodents [27].

This study is the first epidemiological investigation of infectious agents carried by rodents in Franceville and thus contributes to the identification and taxonomic description of infectious agents circulating in Gabon. It highlights the presence of 7 kinds of infectious agents, including several pathogenic agents, particularly *Coxiella burnetii*, *Leptospira* spp., *Bartonella elizabethae* and *Toxoplasma gondii* in Gabonese rodents native to the forest and the savannah rodents *Lophuromys* sp, *Le*. *striatus* and *Praomys* sp. as well as the invasive rodent *R*. *rattus*. These results show that many infectious agents that are pathogenic to humans are in circulation and reveal the need for systematic detection methods for these infectious agents in humans. Indeed, in Africa, many febrile diseases of unknown etiologies can be attributed to these agents. Our results also reveal the need for further studies to establish the zoonotic risks associated with these new potential species of circulating pathogens, particularly Uncultured *Anaplasma* spp, *Candidatus* Bartonella gabonense and *Theileria* sp., to determine whether these agents (new and already known) could be responsible for human cases of febrile diseases of unknown etiology in Gabon.

## Supporting information

S1 Table Positive DNA and RNA controls used in this study. (DOCX)

S2 Table Summary of the total number of rodents captured and prevalence of infections in each district. (DOCX)

S3 Table Details of small mammal species sampled in six districts and vegetation areas of the city. (DOCX)

## References

[1] Luis AD, Hayman DTS, O’Shea TJ, Cryan PM, Gilbert AT, Pulliam JRC, et al. A comparison of bats and rodents as reservoirs of zoonotic viruses: are bats special? Proc R Soc B Biol Sci. 2013;280(1756). 10.1098/rspb.2012.2753 23378666 PMC3574368

[2] Meerburg BG, Singleton GR, Kijlstra A. Rodent-borne diseases and their risks for public health. Crit Rev Microbiol. 2009;(November 2008):1–50. 10.1080/10408410802636017 19548807

[3] Mills J. N. The role of rodents in emerging human disease: examples from the hantaviruses and arenaviruses. Ecological. Singleton GR, Hinds LA, Leirs H, Zhang Z, editors. Australian Centre for International Agricultural Research, Canberra, Australia.; 1999. 134–160 p.

[4] Gratz N. Rodents as carriers of disease. In: Buckle A, Smith R, editors. Rodent pests and their control. Oxford, CAB International; 1994. p. 85.

[5] Keeling MJ, Gilligan CA. Metapopulation dynamics of bubonic plague. Nature. 2000;407(6806):903–6. 10.1038/35038073 11057668

[6] Perry RD, Fetherston JD. Yersinia pestis etiologic agent of plague. Clin Microbiol Rev. 1997 1;10(1):35–66. 10.1128/CMR.10.1.35-66.1997 8993858 PMC172914

[7] Olayemi A, Cadar D, Magassouba N, Obadare A, Kourouma F, Oyeyiola A, et al. New Hosts of The Lassa Virus. Sci Rep. 2016 5 3;6(1):1–6. 10.1038/s41598-016-0001-8 27140942 PMC4853722

[8] Jiang H, Du H, Wang LM, Wang PZ, Bai XF. Hemorrhagic fever with renal syndrome: Pathogenesis and clinical picture. Vol. 6, Frontiers in Cellular and Infection Microbiology. Frontiers Media S.A.; 2016. 10.3389/fcimb.2016.00001 PMC4737898 26870699

[9] Rabaan AA, Al-Ahmed SH, Alsuliman SA, Aldrazi FA, Alfouzan WA, Haque S. The rise of pneumonic plague in Madagascar: Current plague outbreak breaks usual seasonal mould. Vol. 68, Journal of Medical Microbiology. Microbiology Society; 2019. p. 292–302. 10.1099/jmm.0.000915 30632956

[10] Health Organisation World. Lassa Fever–Nigeria Disease outbreak news. 2019.

[11] Mofolorunsho KC. Outbreak of lassa fever in nigeria: Measures for prevention and control. Pan Afr Med J. 2016;23:2–4. 10.11604/pamj.2016.23.2.8451 27347299 PMC4907747

[12] Salmón-Mulanovich G, Powell AR, Hartinger-Peña SM, Schwarz L, Bausch DG, Paz-Soldán VA. Community perceptions of health and rodent-borne diseases along the Inter-Oceanic Highway in Madre de Dios, Peru. BMC Public Health. 2016;16(1):1–10. 27506539 10.1186/s12889-016-3420-3 PMC4979164

[13] Diagne CA, Charbonnel N, Henttonen H, Sironen T, Brouat C. Serological Survey of Zoonotic Viruses in Invasive and Native Commensal Rodents in Senegal, West Africa. Vector-Borne Zoonotic Dis [Internet]. 2017;17(10):730–3. Available from: http://online.liebertpub.com/doi/10.1089/vbz.2017.2135 28873024 10.1089/vbz.2017.2135

[14] Dobigny G, Garba M, Tatard C, Loiseau A, Galan M, Kadaouré I, et al. Urban Market Gardening and Rodent-Borne Pathogenic Leptospira in Arid Zones: A Case Study in Niamey, Niger. PLoS Negl Trop Dis [Internet]. 2015;9(10):e0004097. Available from: http://dx.plos.org/10.1371/journal.pntd.0004097 10.1371/journal.pntd.0004097 26437456 PMC4593649

[15] Aliyu A, Amadu L. Urbanization, cities, and health: The challenges to Nigeria—A review. Vol. 16, Annals of African Medicine. Medknow Publications; 2017. p. 149–58. 10.4103/aam.aam_1_17 PMC5676403 29063897

[16] Neiderud C-J. How urbanization affects the epidemiology of emerging infectious diseases. Infect Ecol Epidemiol [Internet]. 2015;5:27060. Available from: http://www.infectionecologyandepidemiology.net/index.php/iee/article/view/27060/xml_6 10.3402/iee.v5.27060 26112265 PMC4481042

[17] Hove M, Ngwerume ET, Muchemwa C. The urban crisis in Sub-Saharan Africa: A threat to human security and sustainable development. Stability. 2013 3 11;2(1).

[18] Alirol E, Getaz L, Stoll B, Chappuis F, Loutan L. Urbanisation and infectious diseases in a globalised world. Lancet Infect Dis [Internet]. 2011;11(2):131–41. Available from: 10.1016/S1473-3099(10)70223-1 21272793 PMC7106397

[19] Cohen B. Urbanization in developing countries: Current trends, future projections, and key challenges for sustainability. Technol Soc. 2006;28(1–2):63–80.

[20] Gratz NG. Urbanization, arthropod and rodent pests and human health. Proc 3rd Int Conf urban pests. 1999;51–8.

[21] Bonner PC, Schmidt WP, Belmain SR, Oshin B, Baglole D, Borchert M. Poor housing quality increases risk of rodent infestation and lassa fever in refugee camps of sierra leone. Am J Trop Med Hyg. 2007;77(1):169–75. 17620650

[22] Feng AYT, Himsworth CG. The secret life of the city rat: A review of the ecology of urban Norway and black rats (Rattus norvegicus and Rattus rattus). Urban Ecosyst. 2014;17(1):149–62.

[23] Ahmed S, Dávila JD, Allen A, Haklay M (MUKI), Tacoli C, Fèvre EM. Does urbanization make emergence of zoonosis more likely? Evidence, myths and gaps. Environ Urban [Internet]. 2019 10 14 [cited 2020 Mar 11];31(2):443–60. Available from: http://journals.sagepub.com/doi/10.1177/0956247819866124 31656370 10.1177/0956247819866124 PMC6798138

[24] Tong M, Hansen A, Hanson-Easey S, Cameron S, Xiang J, Liu Q, et al. Infectious Diseases, Urbanization and Climate Change: Challenges in Future China. Int J Environ Res Public Health [Internet]. 2015;12(9):11025–36. Available from: http://www.mdpi.com/1660-4601/12/9/11025/ 10.3390/ijerph120911025 26371017 PMC4586659

[25] Olayemi A, Obadare A, Oyeyiola A, Igbokwe J, Fasogbon A, Igbahenah F, et al. Arenavirus Diversity and Phylogeography of Mastomys natalensis Rodents, Nigeria. Emerg Infect Dis. 2016;22(4):13–6. 10.3201/eid2204.150155 26982388 PMC4806934

[26] Andrianaivoarimanana V, Piola P, Wagner DM, Rakotomanana F, Maheriniaina V, Andrianalimanana S, et al. Trends of human plague, madagascar, 1998–2016. Emerg Infect Dis. 2019 2 1;25(2):220–8. 10.3201/eid2502.171974 30666930 PMC6346457

[27] Diagne C, Galan M, Tamisier L, D’Ambrosio J, Dalecky A, Bâ K, et al. Ecological and sanitary impacts of bacterial communities associated to biological invasions in African commensal rodent communities. Sci Rep. 2017 12;7(1):14995. 10.1038/s41598-017-14880-1 29101373 PMC5670214

[28] Diagne C, Ribas A, Charbonnel N, Dalecky A, Tatard C, Gauthier P, et al. Parasites and invasions: changes in gastrointestinal helminth assemblages in invasive and native rodents in Senegal. Int J Parasitol. 2016 12;46(13–14):857–69. 10.1016/j.ijpara.2016.07.007 27670366

[29] Dalecky A, Bâ K, Piry S, Lippens C, Diagne C a., Kane M, et al. Range expansion of the invasive house mouse Mus musculus domesticus in Senegal, West Africa: a synthesis of trapping data over three decades, 1983–2014. Mamm Rev [Internet]. 2015;45(3):176–90. Available from: http://doi.wiley.com/10.1111/mam.12043

[30] Brouat C, Duplantier J-M. Host habitat patchiness and the distance decay of similarity among gastro-intestinal nematode communities in two species of Mastomys (southeastern Senegal). Oecologia [Internet]. 2007 7 [cited 2014 Dec 17];152(4):715–20. Available from: http://www.ncbi.nlm.nih.gov/pubmed/17351796 10.1007/s00442-007-0680-8 17351796

[31] Brouat C, Loiseau A, Kane M, Bâ K, Duplantier J-M. Population genetic structure of two ecologically distinct multimammate rats: the commensal Mastomys natalensis and the wild Mastomys erythroleucus in southeastern Senegal. Mol Ecol [Internet]. 2007 7 [cited 2014 Oct 12];16(14):2985–97. Available from: http://www.ncbi.nlm.nih.gov/pubmed/17614912 10.1111/j.1365-294X.2007.03353.x 17614912

[32] Brouat C, Kane M, Diouf M, Ba K, Sall-Drame R, Duplantier J-M. Host ecology and variation in helminth community structure in Mastomys rodents from Senegal. Parasitology. 2006; 10.1017/S003118200600151X 17076921

[33] Duplantier J-M, Sene M. 24 Rodents as definitive hosts of Schistosoma, with special reference to S. mansoni transmission. In: Morand S, Krasnov BR, Poulin R, editors. Micromammals and Macroparasites [Internet]. Springer-V. 2006. p. 527–43. Available from: 10.1007/978-4-431-36025-4_24

[34] Duplantier J-M, Sene M. Rodents as reservoir hosts in the transmission of Schistosoma mansoni in Richard-Toll, Senegal, west Africa. J Helminthol. 2000;74:129–135. 10881283

[35] Duplantier J, Delta S. Swimming ability in six West- African rodent species under laboratory conditions. African Small Mamm. 1994;

[36] Dobigny G, Poirier P, Hima K, Cabaret O, Gauthier P, Tatard C, et al. Molecular survey of rodent-borne Trypanosoma in Niger with special emphasis on T. lewisi imported by invasive black rats. Acta Trop [Internet]. 2011;117(3):183–8. Available from: 10.1016/j.actatropica.2010.11.004 21126503

[37] Garba M, Dalecky A, Kadaoure I, Kane M, Hima K, Veran S, et al. Spatial Segregation between Invasive and Native Commensal Rodents in an Urban Environment: A Case Study in Niamey, Niger. PLoS One [Internet]. 2014;9(11):e110666. Available from: http://dx.plos.org/10.1371/journal.pone.0110666 10.1371/journal.pone.0110666 25379785 PMC4224371

[38] Tatard C, Garba M, Gauthier P, Hima K, Artige E, Dossou DKHJ, et al. Rodent-borne Trypanosoma from cities and villages of Niger and Nigeria: A special role for the invasive genus Rattus? Acta Trop [Internet]. 2017;171:151–8. Available from: 10.1016/j.actatropica.2017.03.027 28373037

[39] Schwan TG, Lopez JE, Safronetz D, Anderson JM, Fischer RJ, Maïga O, et al. Fleas and trypanosomes of peridomestic small mammals in sub-Saharan Mali. Parasit Vectors [Internet]. 2016 12 11 [cited 2020 Apr 1];9(1):541. Available from: http://parasitesandvectors.biomedcentral.com/articles/10.1186/s13071-016-1818-5 27724960 10.1186/s13071-016-1818-5 PMC5057378

[40] Houemenou G, Ahmed A, Libois R, Hartskeerl RA. Leptospira spp. Prevalence in Small Mammal Populations in Cotonou, Benin. ISRN Epidemiol. 2013;2013:1–8.

[41] Houemenou G, Libois BKR. Ecologie, diversité spécifique et abondance des petits mammifères de la ville de Cotonou au Bénin (Afrique de l ‘ Ouest). Int J Biol Chem Sci. 2014;8(3):1202–13.

[42] Dobigny G, Gauthier P, Houéménou G, Dossou HJ, Badou S, Etougbétché J, et al. Spatio-temporal survey of small mammal-borne Trypanosoma lewisi in Cotonou, Benin, and the potential risk of human infection. Infect Genet Evol. 2019;75(July). 10.1016/j.meegid.2019.103967 31344489

[43] Hima K, Houémenou G, Badou S, Garba M, Dossou HJ, Etougbétché J, et al. Native and invasive small mammals in urban habitats along the commercial axis connecting Benin and Niger, West Africa. Diversity. 2019;11(12):1–20.

[44] Houéménou Gauthier, Etougbeétché Badou, Dossou Agossou, et al. Pathogenic Leptospira in Commensal Small Mammals from the Extensively Urbanized Coastal Benin. Urban Sci. 2019;3(3):99.

[45] Bourgarel M, Wauquier N, Gonzalez JP. Emerging viral threats in Gabon: Health capacities and response to the risk of emerging zoonotic diseases in Central Africa—Emerging zoonotic viral threats in Gabon. Emerg Health Threats J. 2010;3(1). 10.3134/ehtj.10.163 22460397 PMC3167654

[46] Mercier A, Devillard S, Ngoubangoye B, Bonnabau H, Banule A-L, Durand P, et al. Additional Haplogroups of Toxoplasma gondii out of Africa: Population Structure and Mouse-Virulence of Strains from Gabon. PLoS Negl Trop Dis. 2010;4(11):e876. 10.1371/journal.pntd.0000876 21072237 PMC2970538

[47] Richard-Lenoble D, Kombila M, Chandenier J, Engohan E, Gannier M, Dubourg C. Malaria in Gabon I. Study of 500 children with fever in Libreville. Bull Soc Pathol Exot Filiales. 1986 1 1;79(2):284–7. 3524881

[48] Richard-Lenoble D, Kombila M, Chandenier J, Gay F, Billiault X, Nguiri C, et al. Malaria in Gabon 2. Evaluation of the qualitative and quantitative prevalence of parasites in the total school and preschool population of the country. Bull Soc Pathol Exot Filiales. 1987 1 1;80:532–42. 3690800

[49] Borrmann S, Binder RK, Adegnika AA, Missinou MA, Issifou S, Ramharter M, et al. Reassessment of the resistance of Plasmodium falciparum to chloroquine in Gabon: implications for the validity of tests in vitro vs. in vivo. Trans R Soc Trop Med Hyg [Internet]. 2002 11 1 [cited 2020 Apr 30];96(6):660–3. Available from: https://academic.oup.com/trstmh/article-lookup/doi/10.1016/S0035-9203(02)90345-7 12625146 10.1016/s0035-9203(02)90345-7

[50] Bouyou-Akotet MK, Mawili-Mboumba DP, Kendjo E, Mabika-Mamfoumbi M, Ngoungou EB, Dzeing-Ella A, et al. Evidence of decline of malaria in the general hospital of Libreville, Gabon from 2000 to 2008. Malar J. 2009;8(1):300. 10.1186/1475-2875-8-300 20017905 PMC2806380

[51] Assele V, Ndoh GE, Nkoghe D, Fandeur T. No evidence of decline in malaria burden from 2006 to 2013 in a rural Province of Gabon: implications for public health policy. BMC Public Health [Internet]. 2015;15(1):1–8. Available from: http://www.biomedcentral.com/1471-2458/15/81 25649228 10.1186/s12889-015-1456-4 PMC4324784

[52] Mombo IM, Suquet E, Boundenga L, Mveang-Nzoghe A, Maganga-Mboga C, Arnathau C, et al. Detection of novel astroviruses among rodents of Gabon, Central Africa. Infect Genet Evol. 2019 3;68:43–6. 10.1016/j.meegid.2018.12.003 30529088

[53] N′Dilimabaka N, Berthet N, Rougeron V, Mangombi JB, Durand P, Maganga GD, et al. Evidence of Lymphocytic Choriomeningitis Virus (LCMV) in Domestic Mice in Gabon: Risk of Emergence of LCMV Encephalitis in Central Africa. J Virol [Internet]. 2015;89(2):1456–60. Available from: http://jvi.asm.org/lookup/doi/10.1128/JVI.01009-14 25378495 10.1128/JVI.01009-14 PMC4300659

[54] Boundenga L, Ngoubangoye B, Ntie S, Moukodoum N-D, Renaud F, Rougeron V, et al. Rodent malaria in Gabon: Diversity and host range. Int J Parasitol Parasites Wildl. 2019 12;10:117–24. 10.1016/j.ijppaw.2019.07.010 31453086 PMC6702409

[55] Laporte N. Géographie des Relations Ville—Forêt en Afrique Centrale: Approche Régionale (Volume II) [Internet]. Vol. II, Rapport au Biodiversité Support Program, Whashington DC, Décembre 16, 1999. 1999. Available from: http://www.befac.net/pdf/Report-LaPorte1999-AA.pdf%5Cnhttp://carpe.umd.edu/Documents/1999/report-laporte1999-e.pdf%5Cnhttp://carpe.umd.edu/Documents/1999/report-laporte1999-b.pdf

[56] website of the municipality of Franceville. http://www.franceville.ga/. 2012.

[57] Direction de la statistique. Recensement général de la population et des habitats de 2013. 2015.

[58] Population Data.net. https://www.populationdata.net/pays/gabon/. 2020.

[59] Mangombi JB, Brouat C, Loiseau A, Banga O, Leroy EM, Bourgarel M, et al. Urban population genetics of the invasive black rats in Franceville, Gabon. J Zool. 2016;299(3):183–90.

[60] Sikes RS, Gannon WL, Mammalogists T animal care and use commitee of the AS of. Guidelines of the American Society of Mammalogists for the use of wild mammals in research. J Mammal. 2011;92(1):235–53. 10.1093/jmammal/gyw078 PMC5909806 29692469

[61] Nicolas V, Schaeffer B, Missoup AD, Kennis J, Colyn M, Denys C, et al. Assessment of three mitochondrial genes (16S, Cytb, CO1) for identifying species in the Praomyini tribe (Rodentia: Muridae). PLoS One [Internet]. 2012 1 [cited 2013 Oct 31];7(5):e36586. Available from: http://www.pubmedcentral.nih.gov/articlerender.fcgi?artid=3344912&tool=pmcentrez&rendertype=abstract 10.1371/journal.pone.0036586 22574186 PMC3344912

[62] Nicolas V, Mboumba J-F, Verheyen E, Denys C, Lecompte E, Olayemi A, et al. Phylogeographic structure and regional history of Lemniscomys striatus (Rodentia: Muridae) in tropical Africa. J Biogeogr [Internet]. 2008 11 [cited 2013 Oct 31];35(11):2074–89. Available from: http://doi.wiley.com/10.1111/j.1365-2699.2008.01950.x

[63] Nicolas V, Colyn M. Seasonal variations in population and community structure of small rodents in a tropical forest of Gabon. NRC Res Press. 2003;1046:1034–46.

[64] Nicolas V. Population structure and reproduction of Heimyscus fumosus in south-western Gabon. Rev Ecol (Terre Vie). 2003;58.

[65] Nicolas V. Geographical distribution and morphometry of Heimyscus fumosus. Rev D’Écologie (Terre Vie). 2003;58.

[66] Nicolas V, Barrière P, Guimondou S, Colyn M. Variabilite structurale des peuplements forestiers de rongeurs (Muridae) et musaraignes (Soricidae) dans les Monts Doudou, Gabon. 2002.

[67] Duplantier J. Les rongeurs myomorphes forestiers du Nord-est du Gabon: structure du peuplement, démographie, domaines vitaux. Rev Ecol (Terre Vie). 1989;44:329–46.

[68] Duplantier J-M. Critères d’identification des principales espèces …. Gabon. 1987.

[69] Duplanrier J. Les rongeurs myomorphes forestiers du nord-est du Gabon: peuplements, utilisation de l’espace et des ressources alimentaires rôle dans la dispersion et la germination des graines. 1982.

[70] Levett PN. Leptospirosis. Clin Microbiol. 2001;14(2):296–326. 10.1128/CMR.14.2.296-326.2001 PMC88975 11292640

[71] Aitichou M, Saleh SS, McElroy AK, Schmaljohn C, Ibrahim MS. Identification of Dobrava, Hantaan, Seoul, and Puumala viruses by one-step real-time RT-PCR. J Virol Methods. 2005 3;124(1–2):21–6. 10.1016/j.jviromet.2004.10.004 15664046

[72] Klempa B, Fichet-calvet E, Lecompte E, Auste B, Aniskin V, Meisel H, et al. Hantavirus in African Wood Mouse, Guinea. Emerg Infect Dis. 2006;12(5):838–40. 10.3201/eid1205.051487 16704849 PMC3374458

[73] Tong S, Chern SWW, Li Y, Pallansch MA, Anderson LJ. Sensitive and broadly reactive reverse transcription-PCR assays to detect novel paramyxoviruses. J Clin Microbiol. 2008 8;46(8):2652–8. 10.1128/JCM.00192-08 18579717 PMC2519498

[74] Lanciotti RS, Kosoy OL, Laven JJ, Velez JO, Lambert AJ, Johnson AJ, et al. Genetic and serologic properties of Zika virus associated with an epidemic, Yap State, Micronesia, 2007. Emerg Infect Dis. 2008 8;14(8):1232–9. 10.3201/eid1408.080287 18680646 PMC2600394

[75] Moureau G, Temmam S, Gonzalez JP, Charrel RN, Grard G, de Lamballerie X. A real-time RT-PCR method for the universal detection and identification of flaviviruses. Vector Borne Zoonotic Dis. 2007;7(4):467–77. 10.1089/vbz.2007.0206 18020965

[76] Dahmani M, Davoust B, Tahir D, Raoult D, Fenollar F, Mediannikov O. Molecular investigation and phylogeny of Anaplasmataceae species infecting domestic animals and ticks in Corsica, France. Parasites and Vectors. 2017 6 23;10(1). 10.1186/s13071-017-2233-2 28645313 PMC5481957

[77] Mediannikov O, Fenollar F. Looking in ticks for human bacterial pathogens. Microb Pathog [Internet]. 2014;77:142–8. Available from: 10.1016/j.micpath.2014.09.008 25229617

[78] Dahmana H, Granjon L, Diagne C, Davoust B, Fenollar F, Mediannikov O. Rodents as Hosts of Pathogens and Related Zoonotic Disease Risk. Pathogens [Internet]. 2020 3 10 [cited 2020 Mar 25];9(3):202. Available from: http://www.ncbi.nlm.nih.gov/pubmed/32164206 10.3390/pathogens9030202 32164206 PMC7157691

[79] Sokhna C, Mediannikov O, Fenollar F, Bassene H, Diatta G, Tall A, et al. Point-of-Care Laboratory of Pathogen Diagnosis in Rural Senegal. PLoS Negl Trop Dis. 2013;7(1). 10.1371/journal.pntd.0001999 23350001 PMC3547848

[80] Glazunova O, Roux V, Freylikman O, Sekeyova Z, Fournous G, Tyczka J, et al. Coxiella burnetii Genotyping. Emerg Infect Dis. 2005;11(8):1211–7. 10.3201/eid1108.041354 16102309 PMC3320512

[81] Smythe LD, Smith IL, Smith GA, Dohnt MF, Symonds ML, Barnett LJ, et al. A quantitative PCR (TaqMan) assay for pathogenic Leptospira spp. BMC Infect Dis. 2002 7 8;2:13. 10.1186/1471-2334-2-13 12100734 PMC117785

[82] Ahmed N, Manjulata Devi S, de los Á Valverde M, Vijayachari P, Machang’u RS, Ellis WA, et al. Multilocus sequence typing method for identification and genotypic classification of pathogenic Leptospira species. Ann Clin Microbiol Antimicrob. 2006;5:28. 10.1186/1476-0711-5-28 17121682 PMC1664579

[83] Subramanian G, Sekeyova Z, Raoult D, Mediannikov O. Multiple tick-associated bacteria in Ixodes ricinus from Slovakia. Ticks Tick Borne Dis [Internet]. 2012 12 [cited 2020 Apr 3];3(5–6):406–10. Available from: http://www.ncbi.nlm.nih.gov/pubmed/23182274 10.1016/j.ttbdis.2012.10.001 23182274

[84] Bittar F, Keita MB, Lagier JC, Peeters M, Delaporte E, Raoult D. Gorilla gorilla gorilla gut: A potential reservoir of pathogenic bacteria as revealed using culturomics and molecular tools. Sci Rep. 2014 11 24;4. 10.1038/srep07174 25417711 PMC4241516

[85] Kara K. Mitchell TP, Passaretti T, Mitchell KK, Huth P, Smith G, Davidson A. Detection of Streptobacillus moniliformis in whole blood by real-time PCR and review of clinical cases 2004–2015 in New York State. J Microbiol Infect Dis. 2017 6 1;7(2):88–92.

[86] Tomaso H, Jacob D, Eickhoff M, Scholz HC, Al Dahouk S, Kattar MM, et al. Preliminary validation of real-time PCR assays for the identification of Yersinia pestis. Clin Chem Lab Med. 2008;46(9):1239–44. 10.1515/CCLM.2008.251 18783342

[87] Jauregui LH, Higgins J, Zarlenga D, Dubey JP, Lunney JK. Development of a Real-Time PCR Assay for Detection of Toxoplasma gondii in Pig and Mouse Tissues. J Clin Microbiol [Internet]. 2001 6 1 [cited 2020 Mar 4];39(6):2065–71. Available from: http://jcm.asm.org/cgi/doi/10.1128/JCM.39.6.2065-2071.2001 11376036 10.1128/JCM.39.6.2065-2071.2001 PMC88090

[88] Medkour H, Varloud M, Davoust B, Mediannikov O. New Molecular Approach for the Detection of Kinetoplastida Parasites of Medical and Veterinary Interest. Microorganisms [Internet]. 2020 3 2 [cited 2020 Mar 4];8(3):356. Available from: https://www.mdpi.com/2076-2607/8/3/356 10.3390/microorganisms8030356 32131458 PMC7143920

[89] Hall TA. BioEdit: a user-friendly biological sequence alignment editor and analysis program for Windows 95/98/NT. Nucleic Acids Symp Ser. 1999;41:95–8.

[90] Kumar S, Stecher G, Tamura K. MEGA7: Molecular Evolutionary Genetics Analysis Version 7.0 for Bigger Datasets. Mol Biol Evol. 2016 7;33(7):1870–4. 10.1093/molbev/msw054 27004904 PMC8210823

[91] R Development Core Team. R: A language and environment for statistical computing. R Foundation for Statistical Computing, Vienna, Austria. 2007; Available from: http://www.r-project.org/.

[92] Bates D, Mächler M, Bolker BM, Walker SC. Fitting linear mixed-effects models using lme4. J Stat Softw. 2015;67(1).

[93] Barton K. Mu-MIn: Multi-model inference. http://R-Forge.R-project.org/projects/mumin/. 2020.

[94] Benevenute JL, Dumler JS, Ogrzewalska M, Roque ALR, Mello VVC, de Sousa KCM, et al. Assessment of a quantitative 5′ nuclease real-time polymerase chain reaction using groEL gene for Ehrlichia and Anaplasma species in rodents in Brazil. Ticks Tick Borne Dis. 2017 6 1;8(4):646–56. 10.1016/j.ttbdis.2017.04.011 28457822

[95] Kumsa B, Socolovschi C, Almeras L, Raoult D, Parola P. Occurrence and genotyping of coxiella burnetii in ixodid ticks in oromia, Ethiopia. Am J Trop Med Hyg [Internet]. 2015 11 4 [cited 2020 Nov 5];93(5):1074–81. Available from: http://www.ajtmh.org/content/journals/10.4269/ajtmh.14-0758 10.4269/ajtmh.14-0758 26392155 PMC4703278

[96] Han BA, Schmidt JP, Bowden SE, Drake JM. Rodent reservoirs of future zoonotic diseases. Proc Natl Acad Sci [Internet]. 2015;112(22):201501598. Available from: http://www.pnas.org/lookup/doi/10.1073/pnas.1501598112%5Cnhttp://www.pnas.org/content/112/22/7039 26038558 10.1073/pnas.1501598112 PMC4460448

[97] Gu W, Miller S, Chiu CY. Clinical Metagenomic Next-Generation Sequencing for Pathogen Detection. Annu Rev Pathol Mech Dis. 2019 1 24;14(1):319–38. 10.1146/annurev-pathmechdis-012418-012751 30355154 PMC6345613

[98] Colautti RI, Ricciardi A, Grigorovich I a., MacIsaac HJ. Is invasion success explained by the enemy release hypothesis? Ecol Lett. 2004;7(8):721–33.

[99] N’dilimabaka N, Mangombi JB, Maganga G, Banga OL, Simo H, Boundenga L, et al. Absence of arenavirus RNA among animal’s samples from potential reservoirs in Gabonof. SPG BioMed. 2020;

[100] Klempa B, Koivogui L, Sylla O, Koulemou K, Auste B, Krüger DH, et al. Serological evidence of human hantavirus infections in Guinea, West Africa. J Infect Dis. 2010;201(7):1031–4. 10.1086/651169 20187741

[101] Saluzzo JF, Digoutte JP, Adam F, Bauer SP, McCormick JB. Serological evidence for Hantaan-related virus infection in rodents and man in Senegal. Trans R Soc Trop Med Hyg. 1985;79:874–5. 10.1016/0035-9203(85)90145-2 2870570

[102] Chitanga S, Simulundu E, Simuunza MC, Changula K, Qiu Y, Kajihara M, et al. First molecular detection and genetic characterization of Coxiella burnetii in Zambian dogs and rodents. Parasites and Vectors. 2018;11(1):2–5. 10.1186/s13071-017-2577-7 29343277 PMC5773031

[103] Theonest NO, Carter RW, Amani N, Doherty SL, Hugho E, Keyyu JD, et al. Molecular detection and genetic characterization of Bartonella species from rodents and their associated ectoparasites from northern Tanzania. PLoS One. 2019;14(10). 10.1371/journal.pone.0223667 31613914 PMC6793857

[104] Dahmani M, Diatta G, Labas N, Diop A, Bassene H, Raoult D, et al. Noncontiguous finished genome sequence and description of Bartonella mastomydis sp. nov. New Microbes New Infect. 2018;25:60–70. 10.1016/j.nmni.2018.03.005 30128156 PMC6098214

[105] Billeter S a, Borchert JN, Atiku L a, Mpanga JT, Gage KL, Kosoy MY. Bartonella species in invasive rats and indigenous rodents from Uganda. Vector Borne Zoonotic Dis [Internet]. 2014 3 [cited 2014 Dec 18];14(3):182–8. Available from: http://www.ncbi.nlm.nih.gov/pubmed/24575846 10.1089/vbz.2013.1375 24575846

[106] Vanderburg S, Rubach MP, Halliday JEB, Cleaveland S, Reddy EA, Crump JA. Epidemiology of Coxiella burnetii Infection in Africa: A OneHealth Systematic Review. PLoS Negl Trop Dis. 2014;8(4). 10.1371/journal.pntd.0002787 24722554 PMC3983093

[107] André MR. Diversity of Anaplasma and Ehrlichia/Neoehrlichia Agents in terrestrial wild carnivores worldwide: Implications for human and domestic animal health and wildlife conservation. Vol. 5, Frontiers in Veterinary Science. Frontiers Media S.A.; 2018. 10.3389/fvets.2018.00293 30533417 PMC6265506

[108] Galal L, Schares G, Stragier C, Vignoles P, Brouat C, Cuny T, et al. Diversity of Toxoplasma gondii strains shaped by commensal communities of small mammals. Int J Parasitol. 2018 12 1;49(3–4):267–75. 10.1016/j.ijpara.2018.11.004 30578812

[109] Angelakis E, Mediannikov O, Socolovschi C, Mouffok N, Bassene H, Tall A, et al. Coxiella burnetii-positive PCR in febrile patients in rural and urban Africa. Int J Infect Dis. 2014 11;28:107–10. 10.1016/j.ijid.2014.05.029 25245003

[110] Socolovschi C, Mediannikov O, Sokhna C, Tall A, Diatta G, Bassene H, et al. Rickettsia felis-associated uneruptive fever, Senegal. Emerg Infect Dis [Internet]. 2010 7 [cited 2013 Nov 28];16(7):1140–2. Available from: http://www.pubmedcentral.nih.gov/articlerender.fcgi?artid=3321914&tool=pmcentrez&rendertype=abstract 10.3201/eid1607.100070 20587190 PMC3321914

[111] Mediannikov O, Fenollar F, Socolovschi C, Diatta G, Bassene H, Molez J-F, et al. Coxiella burnetii in Humans and Ticks in Rural Senegal. Small PL, editor. PLoS Negl Trop Dis [Internet]. 2010 4 6 [cited 2020 Mar 4];4(4):e654. Available from: http://dx.plos.org/10.1371/journal.pntd.0000654 10.1371/journal.pntd.0000654 20386603 PMC2850317

[112] Picardeau M. Virulence of the zoonotic agent of leptospirosis: Still terra incognita? Nat Rev Microbiol. 2017;15(5):297–307. 10.1038/nrmicro.2017.5 28260786

[113] Xu Y, Zhu Y, Wang Y, Chang YF, Zhang Y, Jiang X, et al. Whole genome sequencing revealed host adaptation-focused genomic plasticity of pathogenic Leptospira. Sci Rep. 2016 2 2;6. 10.1038/srep20020 26833181 PMC4735792

[114] Brenner DJ, Kaufmann AF, Sulzer KR, Steigerwalt AG, Rogers FC, Weyant RS. Further determination of DNA relatedness between serogroups and serovars in the family Leptospiraceae with a proposal for Leptospira alexanderi sp. nov. and four new Leptospira genomospecies. Int J Syst Bacteriol [Internet]. 1999 4 [cited 2020 Apr 12];49(2):839–58. Available from: http://www.ncbi.nlm.nih.gov/pubmed/10319510 10.1099/00207713-49-2-839 10319510

[115] Andre-Fontaine G, Aviat F, Thorin C. Waterborne Leptospirosis: Survival and Preservation of the Virulence of Pathogenic *Leptospira* spp. in Fresh Water. Curr Microbiol. 2015 7 8;71(1):136–42. 10.1007/s00284-015-0836-4 26003629

[116] Gharbi M, Mhadhbi M, Darghouth MA. Diagnostic de la theilériose tropicale du bœuf (infection par Theileria annulata) en Afrique du Nord. Rev Med Vet (Toulouse). 2012;163(12):563–71.

[117] Dumler JS, Choi KS, Garcia-Garcia JC, Barat NS, Scorpio DG, Garyu JW, et al. Human granulocytic anaplasmosis and Anaplasma phagocytophilum. Emerg Infect Dis. 2005;11(12):1828–34. 10.3201/eid1112.050898 16485466 PMC3367650

[118] Rosso F, Tagliapietra V, Baráková I, Derdáková M, Konečný A, Hauffe HC, et al. Prevalence and genetic variability of Anaplasma phagocytophilum in wild rodents from the Italian alps. Parasit Vectors. 2017 6 14;10(1):293. 10.1186/s13071-017-2221-6 28615038 PMC5471728

[119] Chastagner A, Moinet M, Perez G, Roy E, McCoy KD, Plantard O, et al. Prevalence of Anaplasma phagocytophilum in small rodents in France. Ticks Tick Borne Dis [Internet]. 2016 7 1 [cited 2020 Apr 14];7(5):988–91. Available from: https://linkinghub.elsevier.com/retrieve/pii/S1877959X16300784 10.1016/j.ttbdis.2016.05.005 27270190

[120] Zhan L, Cao WC, Jiang JF, Zhang XA, Liu YX, Wu XM, et al. Anaplasma phagocytophilum from rodents and sheep, China. Emerg Infect Dis. 2010;16(5):764–8. 10.3201/eid1605.021293 20409364 PMC2953994

[121] Murphy DS, Lee X, Larson SR, Johnson DKH, Loo T, Paskewitz SM. Prevalence and Distribution of Human and Tick Infections with the Ehrlichia muris-Like Agent and Anaplasma phagocytophilum in Wisconsin, 2009–2015. Vector-Borne Zoonotic Dis [Internet]. 2017 4 1 [cited 2020 Apr 15];17(4):229–36. Available from: http://www.ncbi.nlm.nih.gov/pubmed/28055326 10.1089/vbz.2016.2055 28055326

[122] Beck R, Vojta L, Ćurković S, Mrljak V, Margaletić J, Habrun B. Molecular survey of babesia microti in wild rodents in central croatia. Vector-Borne Zoonotic Dis [Internet]. 2011 1 1 [cited 2020 Apr 16];11(1):81–3. Available from: http://www.ncbi.nlm.nih.gov/pubmed/20553109 10.1089/vbz.2009.0260 20553109

[123] Angelakis E, Billeter SA, Breitschwerdt EB, Chomel BB, Raoult D. Potential for Tick-borne Bartonelloses. Emerg Infect Dis [Internet]. 2010 3 [cited 2020 Apr 14];16(3):385–91. Available from: www.cdc.gov/eid 10.3201/eid1603.081685 20202411 PMC3322042

[124] Parte AC. LPSN—List of prokaryotic names with standing in nomenclature (Bacterio.net), 20 years on. Vol. 68, International Journal of Systematic and Evolutionary Microbiology. Microbiology Society; 2018. p. 1825–9. 10.1099/ijsem.0.002786 29724269

[125] Medkour H, Lo CI, Anani H, Fenollar F, Mediannikov O. Bartonella massiliensis sp. nov., a new bacterial species isolated from an Ornithodoros sonrai tick from Senegal. New Microbes New Infect. 2019 11 1;32. 10.1016/j.nmni.2019.100596 31719993 PMC6839013

[126] Kosoy M, Bai Y, Sheff K, Morway C, Baggett H, Maloney SA, et al. Identification of Bartonella infections in febrile human patients from Thailand and their potential animal reservoirs. Am J Trop Med Hyg. 2010 6 4;82(6):1140–5. 10.4269/ajtmh.2010.09-0778 20519614 PMC2877425

[127] Martin-Alonso A, Houemenou G, Abreu-Yanes E, Valladares B, Feliu C, Foronda P. Bartonella spp. in Small Mammals, Benin. Vector-Borne Zoonotic Dis. 2016;16(4):229–37. 10.1089/vbz.2015.1838 26910412

[128] Barrett MP, Burchmore RJS, Stich A, Lazzari JO, Frasch AC, Cazzulo JJ, et al. The trypanosomiases. In: Lancet. Elsevier Limited; 2003. p. 1469–80. 10.1016/S0140-6736(03)14694-6 14602444

[129] Linardi PM, Botelho JR. Prevalence of Trypanosoma lewisi in Rattus norvegicus from Belo Horizonte, State of Minas Gerais, Brazil. Mem Inst Oswaldo Cruz. 2002;97(3):411–4. 10.1590/s0074-02762002000300024 12048574

[130] Pumhom P, Pognon D, Yangtara S, Thaprathorn N, Milocco C, Douangboupha B, et al. Molecular prevalence of Trypanosoma spp. in wild rodents of Southeast Asia: Influence of human settlement habitat. Epidemiol Infect [Internet]. 2014 6 [cited 2020 Apr 17];142(6):1221–30. Available from: http://www.ncbi.nlm.nih.gov/pubmed/24025128 10.1017/S0950268813002161 24025128 PMC9167665

[131] Truc P, Büscher P, Cuny G, Gonzatti MI, Jannin J, Joshi P, et al. Atypical Human Infections by Animal Trypanosomes. PLoS Negl Trop Dis. 2013;7(9). 10.1371/journal.pntd.0002256 24069464 PMC3772015

[132] Verma A, Manchanda S, Kumar N, Sharma A, Goel M, Banerjee PS, et al. Case report: Trypanosoma lewisi or T. lewisi-like infection in a 37-day-old Indian infant. Am J Trop Med Hyg. 2011 8 1;85(2):221–4. 10.4269/ajtmh.2011.11-0002 21813838 PMC3144816

[133] Nagagi YP, Temba V, Silayo RS, Kweka EJ. Vector-Borne Diseases & Treatment Salient features of Trypanosoma congolense in African Animal Trypanoso- miasis in the sub-Saharan Africa. In: Vector-Borne Diseases & Treatment. 2018.

[134] Reifenberg JM, Solano P, Duvallet G, Cuisance D, Simpore J, Cuny G. Molecular characterization of trypanosome isolates from naturally infected domestic animals in Burkina Faso. Vet Parasitol. 1997;71(4):251–62. 10.1016/s0304-4017(97)00011-3 9299694

[135] Auty HK, Torr SJ, M T, Jayaraman S, Morrison LJ. Cattle trypanosomosis: the diversity of trypanosomes and implications for disease epidemiology and control Trypanosome species of relevance to cattle. Rev sci tech Off int Epiz [Internet]. 2015;4(2):587–98. Available from: www.tritrypdb. 10.20506/rst.34.2.2382 26601459

[136] Rodrigues AC, Ortiz PA, Costa-Martins AG, Neves L, Garcia HA, Alves JMP, et al. Congopain genes diverged to become specific to Savannah, Forest and Kilifi subgroups of Trypanosoma congolense, and are valuable for diagnosis, genotyping and phylogenetic inferences. Infect Genet Evol [Internet]. 2014;23:20–31. Available from: 10.1016/j.meegid.2014.01.012 24480052

[137] Hilton DFJ. Prevalence of trypanosoma otospermophili (protozoa: Trypanosomatidae) in five species of spermophilus (Rodentia: Sciuridae). Parasitology. 1972;65(3):427–32. 10.1017/s0031182000044048 4641489

[138] Mercier A. Approche écologique, épidémiologique et génétique de la biodiversité de Toxoplasma gondii en zone tropicale humide: exemples du Gabon et de la Guyane Française [Internet]. Thèse de doctorat, Université de Limoges; 2010. Available from: http://www.doyoubuzz.com/var/f/fx/At/fxAt4paWh7mXRHe5IgoV-zLq132_uyFJBsNOGSUKcrC6k8YZ0M.pdf

[139] Wang Y, Utzinger J, Saric J, Li J V., Burckhardt J, Dirnhofer S, et al. Global metabolic responses of mice to Trypanosoma brucei brucei infection. Proc Natl Acad Sci U S A. 2008 4 22;105(16):6127–32. 10.1073/pnas.0801777105 18413599 PMC2329718

[140] Cleaveland S, Laurenson MK, Taylor LH. Diseases of humans and their domestic mammals: Pathogen characteristics, host range and the risk of emergence. Philos Trans R Soc B Biol Sci. 2001 7;356(1411):991–9. 10.1098/rstb.2001.0889 PMC1088494 11516377

[141] Kelly D, Paterson R, Townsend C, Poulin R, Tompkins D. Parasite spillback: A neglected concept in invasion ecology? Ecology. 2009;90(8):2047–56. 10.1890/08-1085.1 19739367

[142] Mills JN, Childs JE. Ecologic studies of rodent reservoirs: Their relevance for human health. Emerg Infect Dis. 1998;4(4):529–37. 10.3201/eid0404.980403 9866729 PMC2640244

[143] Boyer N, Réale D, Marmet J, Pisanu B, Chapuis J-L. Personality, space use and tick load in an introduced population of Siberian chipmunks *Tamias sibiricus*. J Anim Ecol [Internet]. 2010 5 1 [cited 2020 Apr 18];79(3):538–47. Available from: http://doi.wiley.com/10.1111/j.1365-2656.2010.01659.x 20202009 10.1111/j.1365-2656.2010.01659.x

