# Supplementary material for: First investigation of pathogenic bacteria, protozoa and viruses in rodents and shrews in context of forest-savannah-urban areas interface in the city of Franceville (Gabon)
Source: PLoS One. 2021 Mar 8;16(3):e0248244. doi: 10.1371/journal.pone.0248244 (PMC7939261; doi:10.1371/journal.pone.0248244)
Supplement: S1 Table — (DOCX) [file pone.0248244.s001.docx]

S1 Table. Positive DNA and RNA controls used in this study.

| Microorganism | Name | Origin |
| --- | --- | --- |
| Bacteria | *Anaplasma phagocytophilum* | Laboratory colony IHU |
| Bacteria | *Bartonella quintana* | Laboratory colony IHU |
| Bacteria | *Bartonella* sp | Laboratory colony IHU |
| Bacteria | *Borrelia crocidurae* | Laboratory colony IHU |
| Bacteria | *Borrrelia* sp | Laboratory colony IHU |
| Bacteria | *Coxiella burnetii* | Laboratory colony IHU |
| Bacteria | *Rickettsia australis* | Laboratory colony IHU |
| Bacteria | *Rickettsia canadensis* | Laboratory colony IHU |
| Bacteria | *Rickettsia conorii* | Laboratory colony IHU |
| Bacteria | *Rickettsia felis* | Laboratory colony IHU |
| Bacteria | *Streptobacillus moniliformis* | Laboratory colony IHU |
| Bacteria | *Wolbachia* | Laboratory colony IHU |
| Bacteria | *Yersinia pestis* | Laboratory colony IHU |
| Parasite | *Leishmania* | Laboratory colony IHU |
| Parasite | *Leishmania major* | Laboratory colony IHU |
| Parasite | *Trypanosoma congolense* | Laboratory colony IHU |
| Parasite | *Trypanosoma gambiense* | Laboratory colony IHU |
| Parasite | *Toxoplasma gondi* | Positive patient DNA, IHU laboratory |
| Parasite | *Piroplasms* | DNA from positive rodents for piroplasms [1] |
| Virus | *Arenavirus* | Plasmid designed in the PREDICT project |
| Virus | *Hantavirus* | Plasmid designed in the PREDICT project |
| Virus | *Flavivirus* | Yellow fever virus RNA (vaccinal strain 17D) |
| Virus | *Paramyxovirus* | RMH* system: Measles virus RNA transcript |
|  |  | AR* system: Mumps virus RNA transcript |
|  |  | PNE* system: Respiratory syncytial virus RNA transcript |

*RMH*:  Respiro*-*, Morbilli*- and *Henipa*- *viruses*; AR: *Avulavirus/Rubulavirus*; PNE: *Pneumovirus*
