# Supplementary material for: First investigation of pathogenic bacteria, protozoa and viruses in rodents and shrews in context of forest-savannah-urban areas interface in the city of Franceville (Gabon)
Source: PLoS One. 2021 Mar 8;16(3):e0248244. doi: 10.1371/journal.pone.0248244 (PMC7939261; doi:10.1371/journal.pone.0248244)
Supplement: S2 Table — (DOCX) [file pone.0248244.s002.docx]

**S2 Table.** Summary of the total number of rodents captured and prevalence of infections in each district.

|  |  | **Central districts** | | **Peripheral districts** | | | | **Vegetative areas** | Infected rodents (prevalence % by species) |
| --- | --- | --- | --- | --- | --- | --- | --- | --- | --- |
|  |  | Potos | Ombélé | Mbaya | Mangoungou | Sable | Yéné | Forest-savannah |  |
| *Rodentia* | *Cricetomys* sp | 0 | 0 | 1 | 1 | 1 | 0 | 0 | 2/3 (66,6) |
|  | *Lemniscomys striatus* | 1 | 2 | 3 | 1 | 2 | 2 | 16 | 14/27 (55,6) |
|  | *Lophuromys sikapusi* | 0 | 0 | 2 | 0 | 1 | 4 | 20 | 17/27(62,9) |
|  | *Mus m. domesticus* | 2 | 0 | 27 | 0 | 0 | 0 | 0 | 1/29 (3,4) |
|  | *Mus Nannonys*sp | 0 | 3 | 4 | 2 | 5 | 6 | 2 | 2/22 (9,09) |
|  | *Praomys* sp | 0 | 1 | 1 | 1 | 3 | 1 | 10 | 4/17 (23,5) |
|  | *Rattus rattus* | 10 | 11 | 10 | 11 | 10 | 2 | 0 | 10/53 (18,9) |
| *Soricomorpha* | *Crocidura goliath* | 2 | 2 | 0 | 3 | 3 | 2 | 1 | 2/13 (15,4) |
|  | *Crocidura poensis* | 0 | 0 | 1 | 0 | 0 | 0 | 3 | 0/4 |
|  | *Sylvisorex ollula* | 0 | 0 | 0 | 0 | 0 | 1 | 0 | 0/1 |
|  | *Crocidura hildegardeae* | 0 | 0 | 0 | 0 | 0 | 0 | 1 | 0/1 |
|  | **Total** | 15 | 19 | 49 | 19 | 25 | 18 | 53 | - |
|  | **Infected rodents (prevalence % by districts)** | **5/15 (33,3)** | **5/19 (26,3)** | **2/49 (4,1)** | **7/19**  **(36,8)** | **5/25 (20)** | **3/18 (16,7)** | **24/53 (45,3)** |  |

Potos and Ombélé are the **central districts** of the city; Mbaya, Mangoungou, Sable and Yéné are the **peripheral districts**.
