# Supplementary material for: First investigation of pathogenic bacteria, protozoa and viruses in rodents and shrews in context of forest-savannah-urban areas interface in the city of Franceville (Gabon)
Source: PLoS One. 2021 Mar 8;16(3):e0248244. doi: 10.1371/journal.pone.0248244 (PMC7939261; doi:10.1371/journal.pone.0248244)
Supplement: S3 Table — (DOCX) [file pone.0248244.s003.docx]

S3 Table. Details of small mammal species sampled in six districts and vegetation areas of the city. The factor data used for the GLMM statistical tests are included.

| Sample ID | Species | Host status | Organs | Districts | Habitats | Richness | Sex | Weight | Height | Trap location | Season | *Bartonella* | *Coxiella* | *Leptospira* | *Anaplasma* | *Toxoplasma* | *Piroplasma* | *Trypanosoma* |
| --- | --- | --- | --- | --- | --- | --- | --- | --- | --- | --- | --- | --- | --- | --- | --- | --- | --- | --- |
| 14MS1000 | *Crocidura poensis* | Native | Liver | Mbaya | Peripheric | 0 | F | 11,5 | 84 | Oudoor | Rainy | 0 | 0 | 0 | 0 | 0 | 0 | 0 |
| 14MS1001 | *Crocidura goliath* | Native | Liver | Yéné | Peripheric | 0 | F | 14 | 105 | Oudoor | Rainy | 0 | 0 | 0 | 0 | 0 | 0 | 0 |
| 14MS1002 | *Sylvisorex ollula* | Native | Liver | Yéné | Peripheric | 0 | F | 11 | 83 | Indoor | Rainy | 0 | 0 | 0 | 0 | 0 | 0 | 0 |
| 14MS1003 | *Crocidura goliath* | Native | Liver | Yéné | Peripheric | 0 | na | 30 | 105 | Oudoor | Rainy | 0 | 0 | 0 | 0 | 0 | 0 | 0 |
| 14MS1004 | *Crocidura goliath* | Native | Kidney | Mangoungou | Peripheric | 1 | M | 32 | 104 | Oudoor | Rainy | 0 | 0 | 1 | 0 | 0 | 0 | 0 |
| 14MS1005 | *Crocidura goliath* | Native | Liver | Mangoungou | Peripheric | 2 | F | 32 | 104 | Indoor | Rainy | 0 | 0 | 1 | 1 | 0 | 0 | 0 |
| 14MS1006 | *Crocidura goliath* | Native | Liver | Potos | Central | 0 | F | 23 | 110 | Indoor | Rainy | 0 | 0 | 0 | 0 | 0 | 0 | 0 |
| 14MS1007 | *Crocidura goliath* | Native | Liver | Potos | Central | 0 | na | 21 | 113 | Indoor | Rainy | 0 | 0 | 0 | 0 | 0 | 0 | 0 |
| 14MS1008 | *Crocidura poensis* | Native | Liver | Vegetal areas | Vegetal | 0 | F | 16 | 100 | Oudoor | Rainy | 0 | 0 | 0 | 0 | 0 | 0 | 0 |
| 14MS1009 | *Crocidura poensis* | Native | Liver | Vegetal areas | Vegetal | 0 | M | 16 | 99 | Oudoor | Rainy | 0 | 0 | 0 | 0 | 0 | 0 | 0 |
| 14MS1010 | *Crocidura goliath* | Native | Liver | Vegetal areas | Vegetal | 0 | F | 36 | 122 | Oudoor | Rainy | 0 | 0 | 0 | 0 | 0 | 0 | 0 |
| 14MS1012 | *Crocidura goliath* | Native | Liver | Mangoungou | Peripheric | 0 | F | 19 | 112 | Oudoor | Dry | 0 | 0 | 0 | 0 | 0 | 0 | 0 |
| 14MS1014 | *Crocidura goliath* | Native | Liver | Ombélé | Central | 0 | na | 22 | 115 | Indoor | Dry | 0 | 0 | 0 | 0 | 0 | 0 | 0 |
| 14MS1015 | *Crocidura goliath* | Native | Liver | Ombélé | Central | 0 | na | 27 | 122 | Oudoor | Dry | 0 | 0 | 0 | 0 | 0 | 0 | 0 |
| 14MS1016 | *Crocidura goliath* | Native | Liver | Sable | Peripheric | 0 | F | 26 | 118 | Oudoor | Dry | 0 | 0 | 0 | 0 | 0 | 0 | 0 |
| 14MS1017 | *Crocidura hildegardeae* | Native | Liver | Vegetal areas | Vegetal | 0 | M | 27 | 112 | Oudoor | Dry | 0 | 0 | 0 | 0 | 0 | 0 | 0 |
| 14MS1018 | *Crocidura goliath* | Native | Liver | Sable | Peripheric | 0 | M | 13 | 100 | Indoor | Dry | 0 | 0 | 0 | 0 | 0 | 0 | 0 |
| 14MS1019 | *Crocidura goliath* | Native | Liver | Sable | Peripheric | 0 | M | 10 | 69 | Indoor | Dry | 0 | 0 | 0 | 0 | 0 | 0 | 0 |
| 14MS1020 | *Crocidura poensis* | Native | Liver | Vegetal areas | Vegetal | 0 | na | 12 | 90 | Oudoor | Dry | 0 | 0 | 0 | 0 | 0 | 0 | 0 |
| 14RD1013 | *Rattus rattus* | Invasive | Liver | Ombélé | Central | 0 | na | 25 | 105 | Oudoor | Dry | 0 | 0 | 0 | 0 | 0 | 0 | 0 |
| 14RD1414 | *Mus domesticus* | Invasive | Liver | Mbaya | Peripheric | 0 | F | 17 | 87 | Indoor | Rainy | 0 | 0 | 0 | 0 | 0 | 0 | 0 |
| 14RD1415 | *Rattus rattus* | Invasive | Liver | Mbaya | Peripheric | 0 | F | 68 | 144 | Indoor | Rainy | 0 | 0 | 0 | 0 | 0 | 0 | 0 |
| 14RD1416 | *Rattus rattus* | Invasive | Liver | Mbaya | Peripheric | 0 | F | 109 | 163 | Indoor | Rainy | 0 | 0 | 0 | 0 | 0 | 0 | 0 |
| 14RD1417 | *Rattus rattus* | Invasive | Liver | Mbaya | Peripheric | 0 | F | 154 | 172 | Indoor | Rainy | 0 | 0 | 0 | 0 | 0 | 0 | 0 |
| 14RD1418 | *Mus domesticus* | Invasive | Liver | Mbaya | Peripheric | 0 | M | 13 | 86 | Indoor | Rainy | 0 | 0 | 0 | 0 | 0 | 0 | 0 |
| 14RD1419 | *Rattus rattus* | Invasive | Liver | Mbaya | Peripheric | 0 | F | 144 | 186 | Oudoor | Rainy | 0 | 0 | 0 | 0 | 0 | 0 | 0 |
| 14RD1420 | *Rattus rattus* | Invasive | Liver | Mbaya | Peripheric | 0 | F | 145 | 178 | Indoor | Rainy | 0 | 0 | 0 | 0 | 0 | 0 | 0 |
| 14RD1422 | *Mus Nannomys* | Native | Liver | Mbaya | Peripheric | 0 | F | 9 | 72 | Oudoor | Rainy | 0 | 0 | 0 | 0 | 0 | 0 | 0 |
| 14RD1423 | *Mus domesticus* | Invasive | Liver | Mbaya | Peripheric | 0 | M | 15 | 89 | Indoor | Rainy | 0 | 0 | 0 | 0 | 0 | 0 | 0 |
| 14RD1425 | *Mus domesticus* | Invasive | Liver | Mbaya | Peripheric | 0 | M | 14 | 86 | Indoor | Rainy | 0 | 0 | 0 | 0 | 0 | 0 | 0 |
| 14RD1429 | *Mus domesticus* | Invasive | Liver | Mbaya | Peripheric | 0 | F | 10 | 86 | Indoor | Rainy | 0 | 0 | 0 | 0 | 0 | 0 | 0 |
| 14RD1430 | *Mus domesticus* | Invasive | Liver | Mbaya | Peripheric | 0 | F | 17 | 103 | Indoor | Rainy | 0 | 0 | 0 | 0 | 0 | 0 | 0 |
| 14RD1431 | *Mus domesticus* | Invasive | Liver | Mbaya | Peripheric | 0 | M | 15 | 86 | Indoor | Rainy | 0 | 0 | 0 | 0 | 0 | 0 | 0 |
| 14RD1434 | *Lemniscomys striatus* | Native | Liver | Mbaya | Peripheric | 0 | M | 41 | 134 | Indoor | Rainy | 0 | 0 | 0 | 0 | 0 | 0 | 0 |
| 14RD1436 | *Lophuromys sikapusi* | Native | Liver | Mbaya | Peripheric | 0 | M | 76 | 150 | Oudoor | Rainy | 0 | 0 | 0 | 0 | 0 | 0 | 0 |
| 14RD1437 | *Mus domesticus* | Invasive | Liver | Mbaya | Peripheric | 0 | M | 15 | 95 | Indoor | Rainy | 0 | 0 | 0 | 0 | 0 | 0 | 0 |
| 14RD1438 | *Mus Nannomys* | Native | Liver | Mbaya | Peripheric | 0 | M | 8 | 65 | Oudoor | Rainy | 0 | 0 | 0 | 0 | 0 | 0 | 0 |
| 14RD1440 | *Praomys sp* | Native | Liver | Mbaya | Peripheric | 0 | M | 31 | 126 | Oudoor | Rainy | 0 | 0 | 0 | 0 | 0 | 0 | 0 |
| 14RD1441 | *Lophuromys sikapusi* | Native | Liver | Mbaya | Peripheric | 0 | M | 85 | 155 | Indoor | Rainy | 0 | 0 | 0 | 0 | 0 | 0 | 0 |
| 14RD1446 | *Mus domesticus* | Invasive | Liver | Mbaya | Peripheric | 1 | M | 12 | 75 | Indoor | Rainy | 0 | 0 | 0 | 0 | 0 | 0 | 1 |
| 14RD1448 | *Rattus rattus* | Invasive | Liver | Yéné | Peripheric | 0 | F | 123 | 174 | Indoor | Rainy | 0 | 0 | 0 | 0 | 0 | 0 | 0 |
| 14RD1451 | *Mus Nannomys* | Native | Liver | Yéné | Peripheric | 0 | F | 8 | 68 | Oudoor | Rainy | 0 | 0 | 0 | 0 | 0 | 0 | 0 |
| 14RD1454 | *Lophuromys sikapusi* | Native | Liver | Yéné | Peripheric | 0 | F | 63 | 37 | Oudoor | Rainy | 0 | 0 | 0 | 0 | 0 | 0 | 0 |
| 14RD1455 | *Mus Nannomys* | Native | Liver | Yéné | Peripheric | 0 | F | 6 | 45 | Oudoor | Rainy | 0 | 0 | 0 | 0 | 0 | 0 | 0 |
| 14RD1456 | *Lemniscomys striatus* | Native | Liver | Yéné | Peripheric | 1 | M | 41 | 125 | Oudoor | Rainy | 0 | 0 | 0 | 1 | 0 | 0 | 0 |
| 14RD1458 | *Mus Nannomys* | Native | Liver | Yéné | Peripheric | 0 | M | 6 | 63 | Oudoor | Rainy | 0 | 0 | 0 | 0 | 0 | 0 | 0 |
| 14RD1459 | *Lophuromys sikapusi* | Native | Liver | Yéné | Peripheric | 0 | M | 90 | 151 | Oudoor | Rainy | 0 | 0 | 0 | 0 | 0 | 0 | 0 |
| 14RD1462 | *Lophuromys sikapusi* | Native | Liver | Yéné | Peripheric | 0 | F | 76 | 140 | Oudoor | Rainy | 0 | 0 | 0 | 0 | 0 | 0 | 0 |
| 14RD1463 | *Mus Nannomys* | Native | Liver | Yéné | Peripheric | 0 | M | 5 | 53 | Oudoor | Rainy | 0 | 0 | 0 | 0 | 0 | 0 | 0 |
| 14RD1467 | *Rattus rattus* | Invasive | Liver | Mangoungou | Peripheric | 0 | F | 98 | 166 | Indoor | Rainy | 0 | 0 | 0 | 0 | 0 | 0 | 0 |
| 14RD1468 | *Rattus rattus* | Invasive | Liver | Mangoungou | Peripheric | 0 | M | 70 | 150 | Oudoor | Rainy | 0 | 0 | 0 | 0 | 0 | 0 | 0 |
| 14RD1469 | *Rattus rattus* | Invasive | Liver | Mangoungou | Peripheric | 0 | F | 125 | 179 | Oudoor | Rainy | 0 | 0 | 0 | 0 | 0 | 0 | 0 |
| 14RD1470 | *Praomys sp* | Native | Liver | Mangoungou | Peripheric | 0 | F | 40 | 127 | Indoor | Rainy | 0 | 0 | 0 | 0 | 0 | 0 | 0 |
| 14RD1471 | *Cricetomys sp* | Native | Liver | Mangoungou | Peripheric | 1 | F | 900 | 340 | Oudoor | Rainy | 1 | 0 | 0 | 0 | 0 | 0 | 0 |
| 14RD1472 | *Lemniscomys striatus* | Native | Liver | Mangoungou | Peripheric | 2 | M | 56 | 142 | Oudoor | Rainy | 0 | 0 | 0 | 1 | 0 | 1 | 0 |
| 14RD1473 | *Rattus rattus* | Invasive | Liver | Mangoungou | Peripheric | 0 | M | 163 | 200 | Oudoor | Rainy | 0 | 0 | 0 | 0 | 0 | 0 | 0 |
| 14RD1474 | *Rattus rattus* | Invasive | Liver | Mangoungou | Peripheric | 0 | F | 59 | 142 | Indoor | Rainy | 0 | 0 | 0 | 0 | 0 | 0 | 0 |
| 14RD1477 | *Mus Nannomys* | Native | Liver | Mangoungou | Peripheric | 0 | F | 7 | 65 | Oudoor | Rainy | 0 | 0 | 0 | 0 | 0 | 0 | 0 |
| 14RD1478 | *Lemniscomys striatus* | Native | Brain | Potos | Central | 1 | F | 65 | 137 | Oudoor | Rainy | 0 | 0 | 0 | 0 | 1 | 0 | 0 |
| 14RD1483 | *Rattus rattus* | Invasive | Liver | Potos | Central | 0 | M | 21 | 89 | Indoor | Rainy | 0 | 0 | 0 | 0 | 0 | 0 | 0 |
| 14RD1484 | *Rattus rattus* | Invasive | Liver | Potos | Central | 1 | M | 118 | 182 | Oudoor | Rainy | 0 | 0 | 0 | 1 | 0 | 0 | 0 |
| 14RD1485 | *Rattus rattus* | Invasive | Liver | Potos | Central | 0 | F | 78 | 154 | Indoor | Rainy | 0 | 0 | 0 | 0 | 0 | 0 | 0 |
| 14RD1486 | *Rattus rattus* | Invasive | Liver | Potos | Central | 0 | F | 73 | 151 | Oudoor | Rainy | 0 | 0 | 0 | 0 | 0 | 0 | 0 |
| 14RD1487 | *Rattus rattus* | Invasive | Liver | Potos | Central | 0 | F | 75 | 148 | Indoor | Rainy | 0 | 0 | 0 | 0 | 0 | 0 | 0 |
| 14RD1501 | *Praomys sp* | Native | Liver | Vegetal areas | Vegetal | 1 | F | 67 | 127 | Oudoor | Rainy | 0 | 0 | 0 | 1 | 0 | 0 | 0 |
| 14RD1502 | *Lemniscomys striatus* | Native | Liver | Vegetal areas | Vegetal | 0 | M | 99 | 110 | Oudoor | Rainy | 0 | 0 | 0 | 0 | 0 | 0 | 0 |
| 14RD1503 | *Lemniscomys striatus* | Native | Liver | Vegetal areas | Vegetal | 0 | M | 61 | 137 | Oudoor | Rainy | 0 | 0 | 0 | 0 | 0 | 0 | 0 |
| 14RD1504 | *Praomys sp* | Native | Liver | Vegetal areas | Vegetal | 1 | F | 28 | 103 | Oudoor | Rainy | 0 | 0 | 0 | 1 | 0 | 0 | 0 |
| 14RD1505 | *Lemniscomys striatus* | Native | Liver | Vegetal areas | Vegetal | 0 | F | 95 | 105 | Oudoor | Rainy | 0 | 0 | 0 | 0 | 0 | 0 | 0 |
| 14RD1506 | *Praomys sp* | Native | Liver | Vegetal areas | Vegetal | 0 | M | 19 | 94 | Oudoor | Rainy | 0 | 0 | 0 | 0 | 0 | 0 | 0 |
| 14RD1507 | *Praomys sp* | Native | Liver | Vegetal areas | Vegetal | 0 | F | 18 | 87 | Oudoor | Rainy | 0 | 0 | 0 | 0 | 0 | 0 | 0 |
| 14RD1508 | *Praomys sp* | Native | Liver | Vegetal areas | Vegetal | 0 | F | 22 | 100 | Oudoor | Rainy | 0 | 0 | 0 | 0 | 0 | 0 | 0 |
| 14RD1509 | *Lemniscomys striatus* | Native | Liver | Vegetal areas | Vegetal | 0 | M | 80 | 145 | Oudoor | Rainy | 0 | 0 | 0 | 0 | 0 | 0 | 0 |
| 14RD1510 | *Praomys sp* | Native | Liver | Vegetal areas | Vegetal | 0 | M | 16 | 86 | Oudoor | Rainy | 0 | 0 | 0 | 0 | 0 | 0 | 0 |
| 14RD1511 | *Praomys sp* | Native | Liver | Vegetal areas | Vegetal | 2 | F | 18 | 90 | Oudoor | Rainy | 0 | 1 | *0* | 0 | 0 | 1 | 0 |
| 14RD1512 | *Lemniscomys striatus* | Native | Liver | Vegetal areas | Vegetal | 0 | F | 53 | 132 | Oudoor | Rainy | 0 | 0 | 0 | 0 | 0 | 0 | 0 |
| 14RD1513 | *Lemniscomys striatus* | Native | Liver | Vegetal areas | Vegetal | 1 | M | 31 | 110 | Oudoor | Rainy | 0 | 0 | 0 | 1 | *0* | 0 | 0 |
| 14RD1514 | *Mus Nannomys* | Native | Liver | Vegetal areas | Vegetal | 0 | M | 7 | 62 | Oudoor | Rainy | 0 | 0 | 0 | 0 | 0 | 0 | 0 |
| 14RD1516 | *Rattus rattus* | Invasive | Liver | Sable | Peripheric | 0 | F | 119 | 177 | Indoor | Rainy | 0 | 0 | 0 | 0 | 0 | 0 | 0 |
| 14RD1517 | *Rattus rattus* | Invasive | Liver | Sable | Peripheric | 0 | F | 139 | 182 | Indoor | Rainy | 0 | 0 | 0 | 0 | 0 | 0 | 0 |
| 14RD1517 | *Rattus rattus* | Invasive | Liver | Sable | Peripheric | 0 | F | 139 | 182 | Indoor | Rainy | 0 | 0 | 0 | 0 | 0 | 0 | 0 |
| 14RD1518 | *Rattus rattus* | Invasive | Liver | Sable | Peripheric | 0 | F | 25 | 97 | Indoor | Rainy | 0 | 0 | 0 | 0 | 0 | 0 | 0 |
| 14RD1519 | *Praomys sp* | Native | Liver | Sable | Peripheric | 1 | M | 41 | 127 | Indoor | Rainy | 0 | 0 | 0 | 1 | 0 | 0 | 0 |
| 14RD1520 | *Mus Nannomys* | Native | Liver | Sable | Peripheric | 0 | M | 9 | 70 | Indoor | Rainy | 0 | 0 | 0 | 0 | 0 | 0 | 0 |
| 14RD1521 | *Lemniscomys striatus* | Native | Liver | Sable | Peripheric | 2 | M | 66 | 132 | Oudoor | Rainy | 0 | 1 | *0* | 1 | 0 | 0 | 0 |
| 14RD1522 | *Lemniscomys striatus* | Native | Liver | Vegetal areas | Vegetal | 0 | F | 54 | 133 | Oudoor | Rainy | 0 | 0 | 0 | 0 | 0 | 0 | 0 |
| 14RD1523 | *Rattus rattus* | Invasive | Liver | Sable | Peripheric | 0 | M | 191 | 198 | Indoor | Rainy | 0 | 0 | 0 | 0 | 0 | 0 | 0 |
| 14RD1528 | *Rattus rattus* | Invasive | Liver | Ombélé | Central | 0 | F | 149 | 181 | Indoor | Rainy | 0 | 0 | 0 | 0 | 0 | 0 | 0 |
| 14RD1529 | *Rattus rattus* | Invasive | Liver | Ombélé | Central | 0 | M | 43 | 134 | Indoor | Rainy | 0 | 0 | 0 | 0 | 0 | 0 | 0 |
| 14RD1530 | *Rattus rattus* | Invasive | Liver | Ombélé | Central | 0 | F | 168 | 191 | Indoor | Rainy | 0 | 0 | 0 | 0 | 0 | 0 | 0 |
| 14RD1531 | *Mus Nannomys* | Native | Liver | Sable | Peripheric | 0 | F | 8 | 73 | Oudoor | Rainy | 0 | 0 | 0 | 0 | 0 | 0 | 0 |
| 14RD1533 | *Rattus rattus* | Invasive | Liver | Ombélé | Central | 0 | F | 150 | 190 | Indoor | Rainy | 0 | 0 | 0 | 0 | 0 | 0 | 0 |
| 14RD1534 | *Rattus rattus* | Invasive | Liver | Ombélé | Central | 0 | M | 41 | 117 | Indoor | Rainy | 0 | 0 | 0 | 0 | 0 | 0 | 0 |
| 14RD1537 | *Lemniscomys striatus* | Native | Liver | Vegetal areas | Vegetal | 1 | M | 41 | 118 | Oudoor | Rainy | 0 | 0 | 0 | 1 | 0 | 0 | 0 |
| 14RD1540 | *Praomys sp* | Native | Liver | Sable | Peripheric | 0 | F | 28 | 110 | Oudoor | Rainy | 0 | 0 | 0 | 0 | 0 | 0 | 0 |
| 14RD1541 | *Rattus rattus* | Invasive | Liver | Sable | Peripheric | 0 | F | 125 | 168 | Indoor | Rainy | 0 | 0 | 0 | 0 | 0 | 0 | 0 |
| 14RD1542 | *Praomys sp* | Native | Liver | Sable | Peripheric | 0 | M | 24 | 100 | Indoor | Rainy | 0 | 0 | 0 | 0 | 0 | 0 | 0 |
| 14RD1550 | *Mus Nannomys* | Native | Liver | Ombélé | Central | 1 | F | 7 | 65 | Oudoor | Rainy | 0 | 0 | 0 | 0 | 0 | 0 | 1 |
| 14RD1554 | *Mus Nannomys* | Native | Liver | Yéné | Peripheric | 0 | M | 7 | 62 | Oudoor | Dry | 0 | 0 | 0 | 0 | 0 | 0 | 0 |
| 14RD1557 | *Rattus rattus* | Invasive | Liver | Mangoungou | Peripheric | 3 | F | 52 | 128 | Indoor | Dry | 0 | 1 | *0* | 1 | 0 | 0 | 1 |
| 14RD1560 | *Mus Nannomys* | Native | Liver | Yéné | Peripheric | 2 | F | 5 | 65 | Indoor | Dry | 0 | 1 | *0* | 0 | 0 | 0 | 1 |
| 14RD1562 | *Lemniscomys striatus* | Native | Liver | Yéné | Peripheric | 0 | F | 21 | 93 | Oudoor | Dry | 0 | 0 | 0 | 0 | 0 | 0 | 0 |
| 14RD1563 | *Lophuromys sikapusi* | Native | Liver | Yéné | Peripheric | 0 | F | 95 | 152 | Oudoor | Dry | 0 | 0 | 0 | 0 | 0 | 0 | 0 |
| 14RD1565 | *Rattus rattus* | Invasive | Liver | Mangoungou | Peripheric | 0 | M | 78 | 156 | Indoor | Dry | 0 | 0 | 0 | 0 | 0 | 0 | 0 |
| 14RD1575 | *Rattus rattus* | Invasive | Liver | Mangoungou | Peripheric | 2 | M | 122 | 178 | Oudoor | Dry | 0 | 0 | 0 | 1 | 0 | 0 | 1 |
| 14RD1576 | *Rattus rattus* | Invasive | Liver | Mangoungou | Peripheric | 0 | F | 52 | 132 | Indoor | Dry | 0 | 0 | 0 | 0 | 0 | 0 | 0 |
| 14RD1577 | *Rattus rattus* | Invasive | Liver | Mangoungou | Peripheric | 0 | F | 164 | 190 | Oudoor | Dry | 0 | 0 | 0 | 0 | 0 | 0 | 0 |
| 14RD1578 | *Rattus rattus* | Invasive | Liver | Mangoungou | Peripheric | 0 | M | 154 | 192 | Indoor | Dry | 0 | 0 | 0 | 0 | 0 | 0 | 0 |
| 14RD1579 | *Mus Nannomys* | Native | Liver | Mangoungou | Peripheric | 0 | F | 7 | 68 | Indoor | Dry | 0 | 0 | 0 | 0 | 0 | 0 | 0 |
| 14RD1580 | *Rattus rattus* | Invasive | Liver | Yéné | Peripheric | 1 | M | 154 | 182 | Indoor | Dry | 0 | 0 | 0 | 0 | 0 | 0 | 1 |
| 14RD1581 | *Praomys sp* | Native | Liver | Yéné | Peripheric | 0 | M | 16 | 85 | Oudoor | Dry | 0 | 0 | 0 | 0 | 0 | 0 | 0 |
| 14RD1590 | *Praomys sp* | Native | Liver | Ombélé | Central | 0 | M | 33 | 120 | Oudoor | Dry | 0 | 0 | 0 | 0 | 0 | 0 | 0 |
| 14RD1596 | *Mus Nannomys* | Native | Liver | Ombélé | Central | 0 | M | 8 | 62 | Oudoor | Dry | 0 | 0 | 0 | 0 | 0 | 0 | 0 |
| 14RD1597 | *Lemniscomys striatus* | Native | Liver | Ombélé | Central | 1 | F | 48 | 125 | Oudoor | Dry | 0 | 0 | 0 | 0 | 0 | 0 | 1 |
| 14RD1598 | *Rattus rattus* | Invasive | Liver | Ombélé | Central | 0 | M | 56 | 121 | Oudoor | Dry | 0 | 0 | 0 | 0 | 0 | 0 | 0 |
| 14RD1599 | *Rattus rattus* | Invasive | Liver | Ombélé | Central | 1 | M | 31 | 106 | Indoor | Dry | 0 | 0 | 0 | 0 | 0 | 0 | 1 |
| 14RD1608 | *Rattus rattus* | Invasive | Liver | Potos | Central | 2 | M | 91 | 157 | Oudoor | Dry | 1 | 0 | 0 | 0 | 0 | 0 | 1 |
| 14RD1609 | *Rattus rattus* | Invasive | Liver | Potos | Central | 0 | M | 37 | 120 | Indoor | Dry | 0 | 0 | 0 | 0 | 0 | 0 | 0 |
| 14RD1610 | *Rattus rattus* | Invasive | Liver | Potos | Central | 0 | F | 79 | 153 | Indoor | Dry | 0 | 0 | 0 | 0 | 0 | 0 | 0 |
| 14RD1611 | *Rattus rattus* | Invasive | Liver | Potos | Central | 1 | F | 32 | 110 | Oudoor | Dry | 0 | 0 | 0 | 0 | 0 | 0 | 1 |
| 14RD1612 | *Rattus rattus* | Invasive | Liver | Potos | Central | 1 | F | 43 | 123 | Indoor | Dry | 0 | 0 | 0 | 0 | 0 | 0 | 1 |
| 14RD1614 | *Rattus rattus* | Invasive | Liver | Ombélé | Central | 1 | F | 60 | 130 | Indoor | Dry | 0 | 0 | 0 | 0 | 0 | 0 | 1 |
| 14RD1615 | *Rattus rattus* | Invasive | Liver | Ombélé | Central | 0 | M | 100 | 173 | Indoor | Dry | 0 | 0 | 0 | 0 | 0 | 0 | 0 |
| 14RD1616 | *Lemniscomys striatus* | Native | Liver | Ombélé | Central | 1 | M | 20 | 95 | Oudoor | Dry | 0 | 0 | 0 | 0 | 0 | 0 | 1 |
| 14RD1617 | *Rattus rattus* | Invasive | Liver | Ombélé | Central | 0 | M | 152 | 197 | Oudoor | Dry | 0 | 0 | 0 | 0 | 0 | 0 | 0 |
| 14RD1622 | *Mus domesticus* | Invasive | Liver | Potos | Central | 0 | M | 13 | 75 | Indoor | Dry | 0 | 0 | 0 | 0 | 0 | 0 | 0 |
| 14RD1626 | *Mus domesticus* | Invasive | Liver | Potos | Central | 0 | F | 11 | 76 | Indoor | Dry | 0 | 0 | 0 | 0 | 0 | 0 | 0 |
| 14RD1641 | *Mus Nannomys* | Native | Liver | Ombélé | Central | 0 | M | 6 | 45 | Oudoor | Dry | 0 | 0 | 0 | 0 | 0 | 0 | 0 |
| 14RD1643 | *Cricetomys sp* | Native | Liver | Sable | Peripheric | 2 | F | 600 | 290 | Oudoor | Dry | 1 | 0 | 0 | 0 | 0 | 0 | 1 |
| 14RD1644 | *Rattus rattus* | Invasive | Liver | Sable | Peripheric | 0 | M | 117 | 183 | Indoor | Dry | 0 | 0 | 0 | 0 | 0 | 0 | 0 |
| 14RD1645 | *Rattus rattus* | Invasive | Liver | Sable | Peripheric | 0 | F | 120 | 185 | Indoor | Dry | 0 | 0 | 0 | 0 | 0 | 0 | 0 |
| 14RD1646 | *Rattus rattus* | Invasive | Kidney | Sable | Peripheric | 1 | F | 102 | 167 | Indoor | Dry | 0 | 0 | 1 | 0 | 0 | 0 | 0 |
| 14RD1647 | *Lophuromys sikapusi* | Native | Kidney | Vegetal areas | Vegetal | 1 | F | 87 | 146 | Oudoor | Dry | 0 | 0 | 1 | 0 | 0 | 0 | 0 |
| 14RD1648 | *Lophuromys sikapusi* | Native | Kidney | Vegetal areas | Vegetal | 2 | M | 83 | 147 | Oudoor | Dry | 1 | 0 | 1 | 0 | 0 | 0 | 0 |
| 14RD1649 | *Lophuromys sikapusi* | Native | Kidney | Vegetal areas | Vegetal | 1 | M | 71 | 132 | Oudoor | Dry | 0 | 0 | 1 | 0 | 0 | 0 | 0 |
| 14RD1650 | *Lemniscomys striatus* | Native | Liver | Vegetal areas | Vegetal | 1 | M | 60 | 125 | Oudoor | Dry | 0 | 1 | *0* | 0 | 0 | 0 | 0 |
| 14RD1651 | *Lophuromys sikapusi* | Native | Liver | Vegetal areas | Vegetal | 0 | F | 80 | 151 | Oudoor | Dry | 0 | 0 | 0 | 0 | 0 | 0 | 0 |
| 14RD1652 | *Rattus rattus* | Invasive | Liver | Sable | Peripheric | 0 | M | 17 | 98 | Indoor | Dry | 0 | 0 | 0 | 0 | 0 | 0 | 0 |
| 14RD1655 | *Mus Nannomys* | Native | Liver | Sable | Peripheric | 0 | F | 8 | 65 | Indoor | Dry | 0 | 0 | 0 | 0 | 0 | 0 | 0 |
| 14RD1657 | *Lophuromys sikapusi* | Native | Liver | Vegetal areas | Vegetal | 0 | M | 41 | 113 | Oudoor | Dry | 0 | 0 | 0 | 0 | 0 | 0 | 0 |
| 14RD1658 | *Lophuromys sikapusi* | Native | Liver | Vegetal areas | Vegetal | 0 | M | 66 | 140 | Oudoor | Dry | 0 | 0 | 0 | 0 | 0 | 0 | 0 |
| 14RD1659 | *Lophuromys sikapusi* | Native | Liver | Vegetal areas | Vegetal | 2 | F | 66 | 135 | Oudoor | Dry | 1 | 1 | *0* | 0 | 0 | 0 | 0 |
| 14RD1660 | *Lophuromys sikapusi* | Native | Liver | Vegetal areas | Vegetal | 1 | F | 65 | 135 | Oudoor | Dry | 1 | 0 | 0 | 0 | 0 | 0 | 0 |
| 14RD1661 | *Lemniscomys striatus* | Native | Liver | Vegetal areas | Vegetal | 1 | M | 61 | 132 | Oudoor | Dry | 0 | 1 | *0* | 0 | 0 | 0 | 0 |
| 14RD1662 | *Lophuromys sikapusi* | Native | Liver | Vegetal areas | Vegetal | 1 | M | 28 | 100 | Oudoor | Dry | 1 | 0 | 0 | 0 | 0 | 0 | 0 |
| 14RD1663 | *Lophuromys sikapusi* | Native | Liver | Vegetal areas | Vegetal | 0 | M | 70 | 136 | Oudoor | Dry | 0 | 0 | 0 | 0 | 0 | 0 | 0 |
| 14RD1664 | *Lophuromys sikapusi* | Native | Liver | Vegetal areas | Vegetal | 0 | M | 47 | 118 | Oudoor | Dry | 0 | 0 | 0 | 0 | 0 | 0 | 0 |
| 14RD1665 | *Lophuromys sikapusi* | Native | Liver | Vegetal areas | Vegetal | 0 | M | 87 | 146 | Oudoor | Dry | 0 | 0 | 0 | 0 | 0 | 0 | 0 |
| 14RD1666 | *Lemniscomys striatus* | Native | Liver | Vegetal areas | Vegetal | 1 | M | 67 | 134 | Oudoor | Dry | 0 | 1 | *0* | 0 | 0 | 0 | 0 |
| 14RD1667 | *Lophuromys sikapusi* | Native | Liver | Vegetal areas | Vegetal | 2 | M | 64 | 127 | Oudoor | Dry | 1 | 0 | 0 | 1 | 0 | 0 | 0 |
| 14RD1668 | *Lophuromys sikapusi* | Native | Liver | Vegetal areas | Vegetal | 1 | M | 38 | 112 | Oudoor | Dry | 1 | 0 | 0 | 0 | 0 | 0 | 0 |
| 14RD1669 | *Lophuromys sikapusi* | Native | Liver | Vegetal areas | Vegetal | 1 | F | 48 | 120 | Oudoor | Dry | 1 | 0 | 0 | 0 | 0 | 0 | 0 |
| 14RD1670 | *Lemniscomys striatus* | Native | Liver | Vegetal areas | Vegetal | 1 | M | 64 | 127 | Oudoor | Dry | 0 | 0 | 0 | 1 | 0 | 0 | 0 |
| 14RD1671 | *Mus Nannomys* | Native | Liver | Sable | Peripheric | 0 | M | 6 | 60 | Oudoor | Dry | 0 | 0 | 0 | 0 | 0 | 0 | 0 |
| 14RD1673 | *Lemniscomys striatus* | Native | Liver | Sable | Peripheric | 0 | M | 41 | 120 | Oudoor | Dry | 0 | 0 | 0 | 0 | 0 | 0 | 0 |
| 14RD1674 | *Mus Nannomys* | Native | Liver | Sable | Peripheric | 0 | F | 8 | 67 | Indoor | Dry | 0 | 0 | 0 | 0 | 0 | 0 | 0 |
| 14RD1676 | *Lophuromys sikapusi* | Native | Liver | Sable | Peripheric | 1 | F | 68 | 144 | Oudoor | Dry | 1 | 0 | 0 | 0 | 0 | 0 | 0 |
| 14RD1678 | *Lophuromys sikapusi* | Native | Liver | Vegetal areas | Vegetal | 0 | M | 46 | 118 | Oudoor | Dry | 0 | 0 | 0 | 0 | 0 | 0 | 0 |
| 14RD1679 | *Mus Nannomys* | Native | Liver | Vegetal areas | Vegetal | 0 | M | 6 | 60 | Oudoor | Dry | 0 | 0 | 0 | 0 | 0 | 0 | 0 |
| 14RD1680 | *Lophuromys sikapusi* | Native | Liver | Vegetal areas | Vegetal | 2 | M | 76 | 140 | Oudoor | Dry | 1 | 1 | *0* | 0 | 0 | 0 | 0 |
| 14RD1681 | *Lophuromys sikapusi* | Native | Kidney | Vegetal areas | Vegetal | 1 | F | 81 | 151 | Oudoor | Dry | 0 | 0 | 1 | 0 | 0 | 0 | 0 |
| 14RD1682 | *Lemniscomys striatus* | Native | Liver | Vegetal areas | Vegetal | 1 | M | 45 | 120 | Oudoor | Dry | 0 | 0 | 0 | 1 | 0 | 0 | 0 |
| 14RD1683 | *Lemniscomys striatus* | Native | Liver | Vegetal areas | Vegetal | 0 | F | 48 | 120 | Oudoor | Dry | 0 | 0 | 0 | 0 | 0 | 0 | 0 |
| 14RD1684 | *Lophuromys sikapusi* | Native | Liver | Vegetal areas | Vegetal | 1 | F | 61 | 128 | Oudoor | Dry | 1 | 0 | 0 | 0 | 0 | 0 | 0 |
| 14RD1685 | *Lemniscomys striatus* | Native | Liver | Vegetal areas | Vegetal | 1 | M | 53 | 130 | Oudoor | Dry | 0 | 1 | *0* | 0 | 0 | 0 | 0 |
| 14RD1689 | *Mus domesticus* | Invasive | Liver | Mbaya | Peripheric | 0 | F | 14 | 80 | Indoor | Dry | 0 | 0 | 0 | 0 | 0 | 0 | 0 |
| 14RD1690 | *Mus domesticus* | Invasive | Liver | Mbaya | Peripheric | 0 | M | 13 | 80 | Indoor | Dry | 0 | 0 | 0 | 0 | 0 | 0 | 0 |
| 14RD1691 | *Mus domesticus* | Invasive | Liver | Mbaya | Peripheric | 0 | F | 16 | 89 | Indoor | Dry | 0 | 0 | 0 | 0 | 0 | 0 | 0 |
| 14RD1692 | *Mus domesticus* | Invasive | Liver | Mbaya | Peripheric | 0 | F | 15 | 90 | Indoor | Dry | 0 | 0 | 0 | 0 | 0 | 0 | 0 |
| 14RD1693 | *Mus domesticus* | Invasive | Liver | Mbaya | Peripheric | 0 | F | 13 | 80 | Indoor | Dry | 0 | 0 | 0 | 0 | 0 | 0 | 0 |
| 14RD1694 | *Mus domesticus* | Invasive | Liver | Mbaya | Peripheric | 0 | F | 14 | 85 | Indoor | Dry | 0 | 0 | 0 | 0 | 0 | 0 | 0 |
| 14RD1695 | *Mus Nannomys* | Native | Liver | Mbaya | Peripheric | 0 | F | 6 | 60 | Indoor | Dry | 0 | 0 | 0 | 0 | 0 | 0 | 0 |
| 14RD1699 | *Cricetomys sp* | Native | Liver | Mbaya | Peripheric | 0 | M | 600 | 290 | Oudoor | Dry | 0 | 0 | 0 | 0 | 0 | 0 | 0 |
| 14RD1700 | *Lemniscomys striatus* | Native | Liver | Vegetal areas | Vegetal | 0 | F | 56 | 137 | Oudoor | Dry | 0 | 0 | 0 | 0 | 0 | 0 | 0 |
| 14RD1701 | *Mus domesticus* | Invasive | Liver | Mbaya | Peripheric | 0 | F | 11 | 80 | Indoor | Dry | 0 | 0 | 0 | 0 | 0 | 0 | 0 |
| 14RD1702 | *Mus domesticus* | Invasive | Liver | Mbaya | Peripheric | 0 | F | 12 | 80 | Indoor | Dry | 0 | 0 | 0 | 0 | 0 | 0 | 0 |
| 14RD1703 | *Mus domesticus* | Invasive | Liver | Mbaya | Peripheric | 0 | F | 14 | 80 | Indoor | Dry | 0 | 0 | 0 | 0 | 0 | 0 | 0 |
| 14RD1704 | *Mus Nannomys* | Native | Liver | Mbaya | Peripheric | 0 | M | 4 | 59 | Indoor | Dry | 0 | 0 | 0 | 0 | 0 | 0 | 0 |
| 14RD1705 | *Mus domesticus* | Invasive | Liver | Mbaya | Peripheric | 0 | F | 16 | 86 | Indoor | Dry | 0 | 0 | 0 | 0 | 0 | 0 | 0 |
| 14RD1706 | *Mus domesticus* | Invasive | Liver | Mbaya | Peripheric | 0 | F | 15 | 87 | Indoor | Dry | 0 | 0 | 0 | 0 | 0 | 0 | 0 |
| 14RD1709 | *Lophuromys sikapusi* | Native | Liver | Vegetal areas | Vegetal | 0 | M | 57 | 133 | Oudoor | Dry | 0 | 0 | 0 | 0 | 0 | 0 | 0 |
| 14RD1710 | *Praomys sp* | Native | Liver | Vegetal areas | Vegetal | 0 | F | 9 | 72 | Oudoor | Dry | 0 | 0 | 0 | 0 | 0 | 0 | 0 |
| 14RD1711 | *Rattus rattus* | Invasive | Liver | Mbaya | Peripheric | 0 | M | 133 | 193 | Indoor | Dry | 0 | 0 | 0 | 0 | 0 | 0 | 0 |
| 14RD1712 | *Rattus rattus* | Invasive | Liver | Mbaya | Peripheric | 0 | F | 61 | 150 | Oudoor | Dry | 0 | 0 | 0 | 0 | 0 | 0 | 0 |
| 14RD1713 | *Rattus rattus* | Invasive | Liver | Mbaya | Peripheric | 0 | F | 133 | 180 | Indoor | Dry | 0 | 0 | 0 | 0 | 0 | 0 | 0 |
| 14RD1714 | *Mus domesticus* | Invasive | Liver | Mbaya | Peripheric | 0 | M | 14 | 80 | Indoor | Dry | 0 | 0 | 0 | 0 | 0 | 0 | 0 |
| 14RD1715 | *Mus domesticus* | Invasive | Liver | Mbaya | Peripheric | 0 | M | 12 | 83 | Indoor | Dry | 0 | 0 | 0 | 0 | 0 | 0 | 0 |
| 14RD1716 | *Mus domesticus* | Invasive | Liver | Mbaya | Peripheric | 0 | F | 13 | 80 | Indoor | Dry | 0 | 0 | 0 | 0 | 0 | 0 | 0 |
| 14RD1717 | *Mus domesticus* | Invasive | Liver | Mbaya | Peripheric | 0 | F | 14 | 82 | Indoor | Dry | 0 | 0 | 0 | 0 | 0 | 0 | 0 |
| 14RD1718 | *Mus domesticus* | Invasive | Liver | Mbaya | Peripheric | 0 | M | 5 | 62 | Indoor | Dry | 0 | 0 | 0 | 0 | 0 | 0 | 0 |
| 14RD1719 | *Praomys sp* | Native | Liver | Vegetal areas | Vegetal | 0 | M | 15 | 85 | Oudoor | Dry | 0 | 0 | 0 | 0 | 0 | 0 | 0 |
| 14RD1720 | *Praomys sp* | Native | Liver | Vegetal areas | Vegetal | 0 | M | 19 | 95 | Oudoor | Dry | 0 | 0 | 0 | 0 | 0 | 0 | 0 |
| 14RD1721 | *Lemniscomys striatus* | Native | Liver | Mbaya | Peripheric | 0 | F | 39 | 115 | Indoor | Dry | 0 | 0 | 0 | 0 | 0 | 0 | 0 |
| 14RD1722 | *Mus domesticus* | Invasive | Liver | Mbaya | Peripheric | 0 | M | 5 | 60 | Indoor | Dry | 0 | 0 | 0 | 0 | 0 | 0 | 0 |
| 14RD1723 | *Mus domesticus* | Invasive | Liver | Mbaya | Peripheric | 0 | M | 15 | 83 | Indoor | Dry | 0 | 0 | 0 | 0 | 0 | 0 | 0 |
| 14RD1724 | *Rattus rattus* | Invasive | Liver | Mbaya | Peripheric | 0 | F | 114 | 170 | Indoor | Dry | 0 | 0 | 0 | 0 | 0 | 0 | 0 |
| 14RD1725 | *Rattus rattus* | Invasive | Liver | Mbaya | Peripheric | 0 | F | 136 | 178 | Indoor | Dry | 0 | 0 | 0 | 0 | 0 | 0 | 0 |
| 14RD1726 | *Lemniscomys striatus* | Native | Liver | Mbaya | Peripheric | 1 | F | 57 | 137 | Oudoor | Dry | 0 | 0 | 0 | 1 | 0 | 0 | 0 |
